# SESAME – a synchrotron light source in the Middle East: an international research infrastructure in the making

**DOI:** 10.12688/openreseurope.13362.1

**Published:** 2021-05-17

**Authors:** Charlotte Rungius, Tim Flink, Sebastian Riedel

**Affiliations:** 1 Humboldt University of Berlin, Berlin, Germany; 2 DZHW, Berlin, Germany

**Keywords:** Science Diplomacy, SESAME, Science for Peace, S4D4C

## Abstract

SESAME (Synchrotron-light for Experimental Science and Applications in the Middle East) is an international research centre located in Jordan. The centre was established in the late 1990s with the intention to foster scientific cooperation in a region of the world that has been torn by persistent conflicts. The project is built on the idea that science can help to overcome barriers and cultural differences within the common ground of science and research. SESAME’s core ambition is to operate an international state-of-the-art synchrotron users’ facility in the north of Amman that is accessible to scientists from all of its member countries: Cyprus, Egypt, Iran, Israel, Pakistan, Palestine, Turkey and Jordan. Accordingly, SESAME has been often praised as a paramount example of science diplomacy. Our intention for this report was to investigate a concrete international research infrastructure with a specific science diplomacy interest. What were the enabling conditions for such a project to come into being and what keeps SESAME running? What were the challenges and obstacles? How does the composition of member states play into that? How is SESAME related to (and embedded in) the global synchrotron community, academia, researchers and political actors in the region and the world? Did the science diplomacy ambition behind the project turn out to be successful and how does it affect SESAME's future?

## 1. Introduction

SESAME is short for “Synchrotron-light for Experimental Science and Applications in the Middle East” and is the first synchrotron light source in the Middle East, out of about 60 synchrotrons worldwide. It was developed under the auspices of the United Nations Educational, Scientific and Cultural Organization (UNESCO) and is now a multinational, interdisciplinary and independent research facility located in Allan in the Balqa governorate of Jordan, northwest of the capital Amman towards the Syrian borderline. While the origins of SESAME reach back to the 1990s, the first two beamlines were installed in November 2017 (X-ray spectrum) and in April 2018 (infrared spectrum, IR). The research facility was opened on May 16 ^th^, 2017 and different groups from the region have started to conduct research at the beamlines.

SESAME was established in the late 1990s with the intention to foster scientific cooperation in a region of the world that has been torn by persistent conflicts. The project is built on the idea that science can help to overcome barriers and cultural differences within the common ground of science and research. In that regard, SESAME is an important example for use in studying the general research interests of S4D4C ^1^: How can science diplomacy foster international cooperation and help to tackle global challenges? And what can we learn from the example of SESAME about inducing and making use of research infrastructures for the benefit of international relations, intercultural understanding and economic and technological development within and beyond Europe? From that point of view, SESAME classifies as an example of “science in diplomacy” when taking into account global challenges, mainly in the form of peace and intercultural understanding. Needless to say, this case implicates a good deal of “diplomacy for science” (political activities to support international scientific cooperation) on the concrete level of implementation as well.

Within the terminology of S4D4C, SESAME is considered to be primarily an instrument driven science diplomacy case, as opposed to science driven or foreign policy driven cases. Instrument driven cases refer to science diplomacy configurations that originate in funding mechanisms, science collaborations, or infrastructures. Therefore, this study takes particular interest in the funding structures and the contribution of institutional stakeholders and in that regard especially the role of the EU in comparison to other stakeholders. At the same time, this case also demonstrates aspects of a science driven case. Science driven cases are science diplomacy configurations that originate in scientific or technological developments and as a consequence of their advancement involve and/or affect inter- or transnational cooperation or regulations (open science, FET [future emerging technologies] flagships, specific expertise in a field of research). SESAME’s primary goal is to serve a scientific purpose in the form of a users’ synchrotron facility, and in doing so it involves international actors and requires unique forms of international cooperation. By contrast, foreign policy-driven cases are finally science diplomacy configurations that depart from political intentions or concerns, usually with an international context (climate change, cyber security, and infectious diseases) and as part of that they involve scientific knowledge or advice. Therefore, the role of science in science driven cases is confined to the provision of knowledge to solve or regulate collective problems, which is not the focus of interest in this case.

Given this blending of instrument and science driven science diplomacy configurations, two aspects in particular are of relevance for the general research interest. First, since the structure has a fairly short history and is still in the development phase, the case provides the *opportunity to explore the critical transition phase from vision to reality* more closely. How does a project like SESAME come into being – what is crucial to master the steps from an initial idea to a research site with its own routines and sustainable procedures? Furthermore, and as part of that, we can learn about the intentions and motives behind the initiation of such a project and we can ask: How do they maybe affect the success and further evolution of the project? What are the main drivers and resources that carry such a project from the beginning to continuity and how does this change during the course of its advancement? What was the initial spark and which structures had to be developed to carry the idea into a physical reality? What conditions and circumstances might have been major challenges or obstacles?

Besides this interest in the evolution of SESAME as a fairly unique science diplomacy case, it also provides us with the opportunity to learn about science diplomacy on a more conceptual level. The discussion of science diplomacy is mostly led by rather general and associative understandings of science and diplomacy ^2, 3^. By contrast, SESAME provides a real-world example to study those concrete interactions and practices on the social micro level that we otherwise would broadly summarize and *synthesize* under the label science diplomacy. Therefore, this case study is also suitable to break the conceptions of science and diplomacy within the concept down to the level of individual building bricks. SESAME is a research site designed to bring together scientists from different regions and backgrounds not only in order to conduct research, but also in order to establish contact and communication channels that would otherwise not be possible. In this sense, SESAME provides an *analytical* case study (in contrast to synthetic), which allows our general imagination of science as a means for peace building to be examined and dissected on the “atomic” level of social practice and communication.

Similarly, the research interest in the research infrastructures of this report is tailored to a science diplomacy perspective. It does not provide an understanding of SESAME as a technical facility as such. Therefore, it cannot do justice to the SESAME project in all its dimensions and achievements. Finally, this report is not meant to reproduce the most prominent or dominant narratives about SESAME and to prove them either right or wrong. The story of SESAME often has been told as a story of hope and promising peace building efforts in the Middle East. Yet, our due task is not to pick and choose a certain angle. Neither can we assess the “real” peace building outcomes of SESAME. There is not one single truth to be told about SESAME, there are many. The task here is to illustrate the narratives that constitute the project as a discursive and material reality, to highlight contradictions and variations and finally to work out how the external narratives may interact and impact the inner logic of the research site against the background of our specific science diplomacy research interest. In that, SESAME provides an institutionally clearly demarcated structure to study aspects of science diplomacy in a unique setting and as an example for bottom-up science diplomacy initiatives by scientists. Ultimately, we have observed immense commitment and endurance by an international group of scientists and staff from around the world to establish this research infrastructure under extraordinary conditions and to advance it into a success story. At the same time, it does not come as a surprise that the case of SESAME also absorbs and echoes the conditions and conflicts it came to address and transform ^4^.

## 2. Case study background

### 2.1 Case description and context of the case

**Figure 1.**
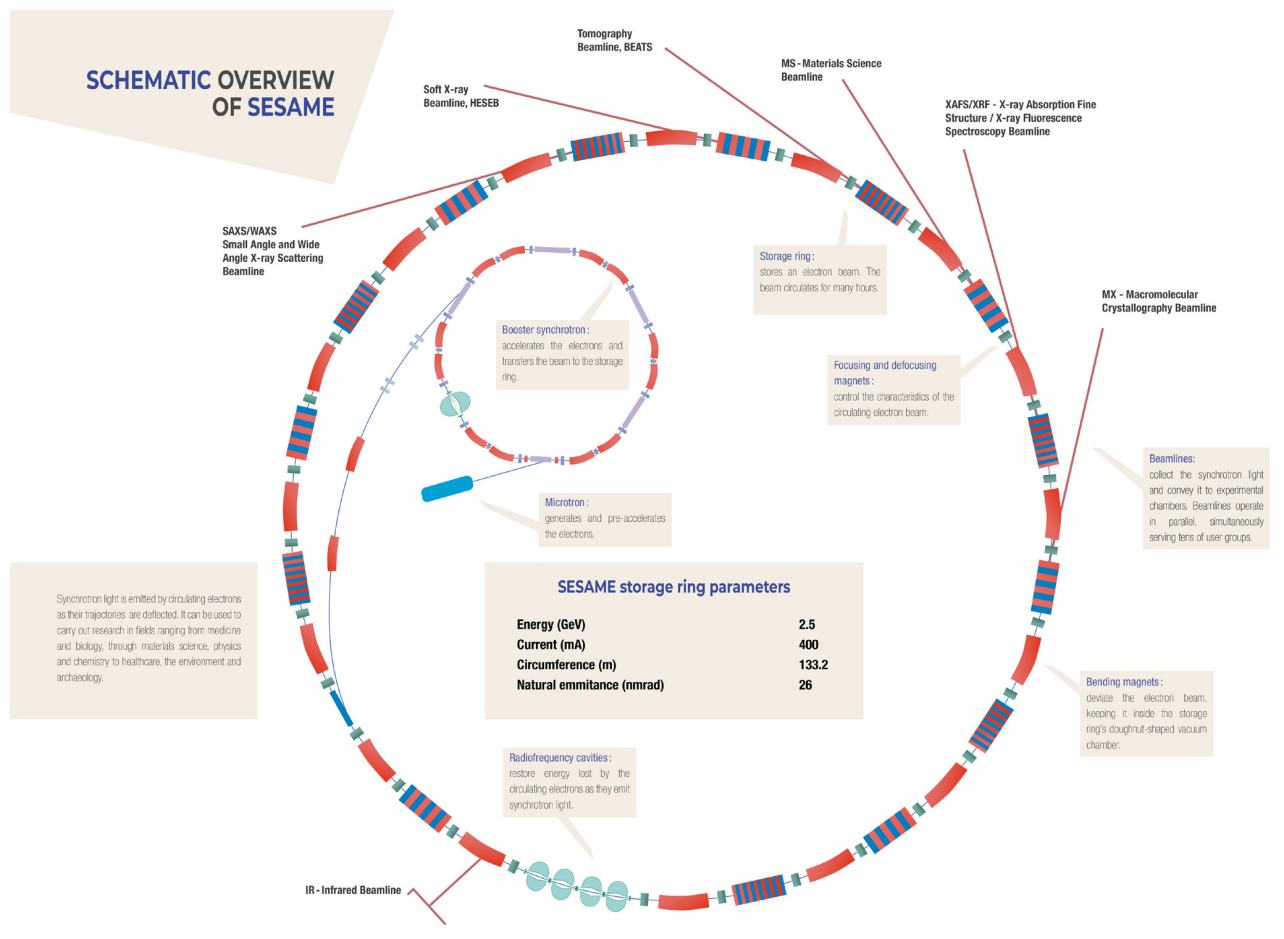
Schematic outline of the SESAME synchrotron facility with storage ring.

**Figure 2.**
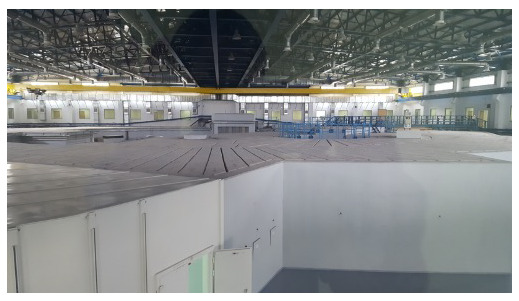
photo of storage ring, December 2018.

SESAME is an international research centre under the auspices of the United Nations Educational, Scientific and Cultural Organization (UNESCO) that runs a third-generation synchrotron radiation facility (in short: *synchrotron*) in Allan, Jordan ^5^. SESAME is the first synchrotron in the Middle East and in an Arab country and the first synchrotron ever that was meant to be composed of modules that were shipped from and reconstructed in different countries ^6^. A synchrotron is a technically highly sophisticated light source that enables the study of the molecular structure of matter such as proteins, crystals or viruses. Synchrotrons have become a substantial, highly automatized state-of-the-art device in order to conduct cutting-edge research in a large variety of disciplines ^7^. Synchrotron radiation user facilities have decisively advanced scientific understanding in the life sciences and material sciences in the last few decades and allow for a great variety of experimental applications at different wavelengths in the spectrum of light ( Figure 1, Figure 2).

A synchrotron is a type of particle accelerator that is built to produce very brilliant light. Synchrotrons were originally constructed for basic research in high-energy physics, but they have become an important and rapidly developing experimental research device in many disciplines. Synchrotron light is employed in many different laboratory applications (e.g. spectroscopy, microscopy, diffraction experiments) across many disciplines and research fields in the natural sciences, namely medicine, biology, material science and archaeology ^8^.

Synchrotron light sources are not only technically sophisticated, but costly large research infrastructures. Therefore these facilities are typically run by public, state-owned or even intergovernmental research (funding) organizations. The vast majority of the approximately 60 currently operational synchrotrons worldwide are located in industrialized countries.

**Figure 3.**
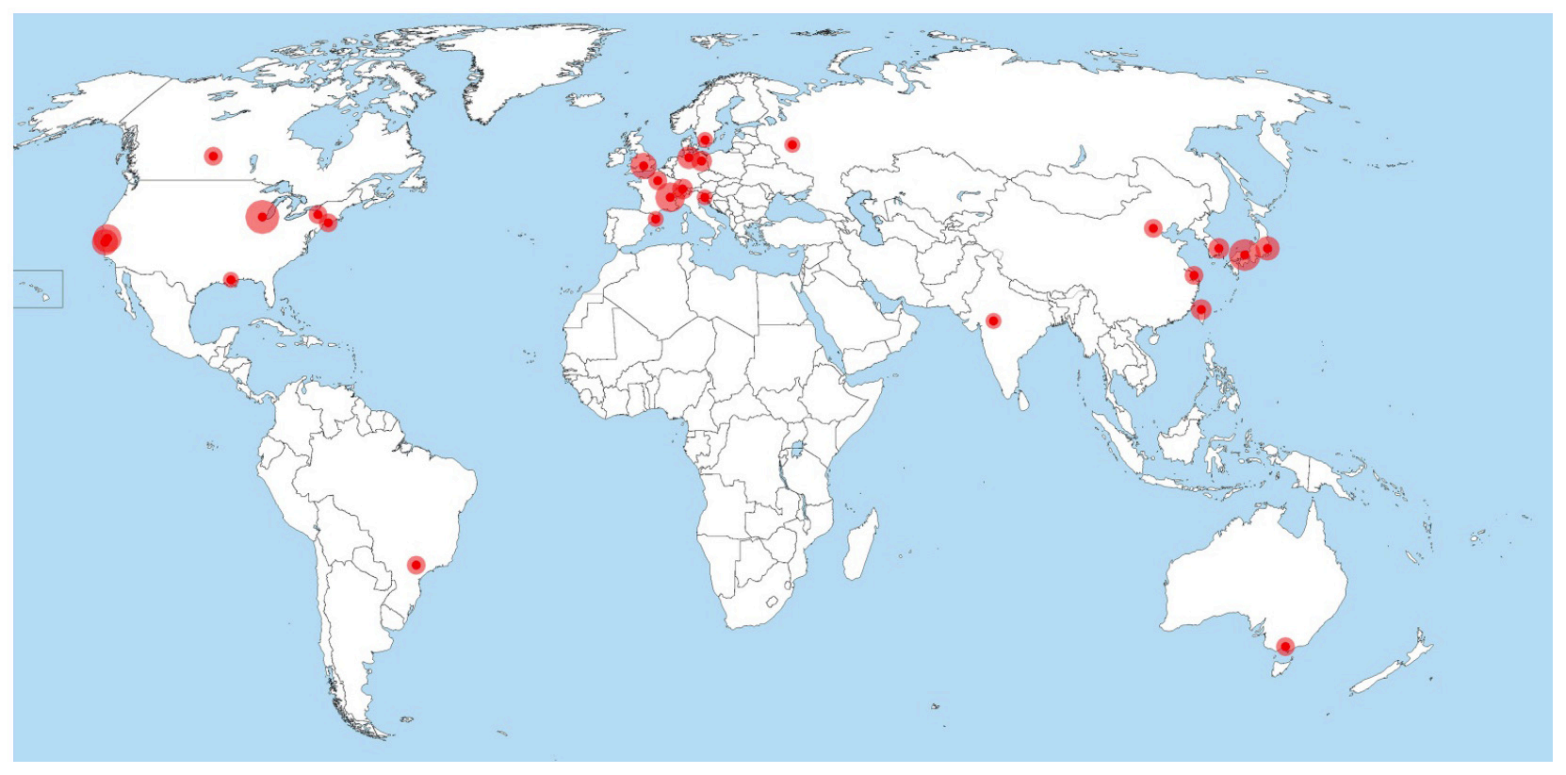
Distribution of synchrotrons with operational MX beamlines worldwide in 2016 ^11^.

The United States alone owns more than 10 synchrotron facilities (with different technical properties and applications, however), so does Japan. The biggest share of facilities worldwide rests in Europe (namely Germany, France, Switzerland, the UK, Italy, Sweden, Russia, Denmark, Netherlands and Spain; see Figure 3 ^9^). Similarly, most industrialized countries have at least one synchrotron radiation user facility or have access to one, while there are very few facilities in the less technologized regions of the world ( Figure 3). Namely Brazil and Taiwan started to design own synchrotrons in the late 1980s and have internationally competitive machines at the time of research (2019) ^10^. They were followed by Singapore, India, Thailand and Poland, who inaugurated their own synchrotrons during the last two decades. Iran and Armenia have announced their intentions to build their own facilities but have not realized these plans so far (see grey pins in Figure 5). Turkey is currently building its first IR-FEL facility (operating in the infrared-spectrum).

**Figure 4.**
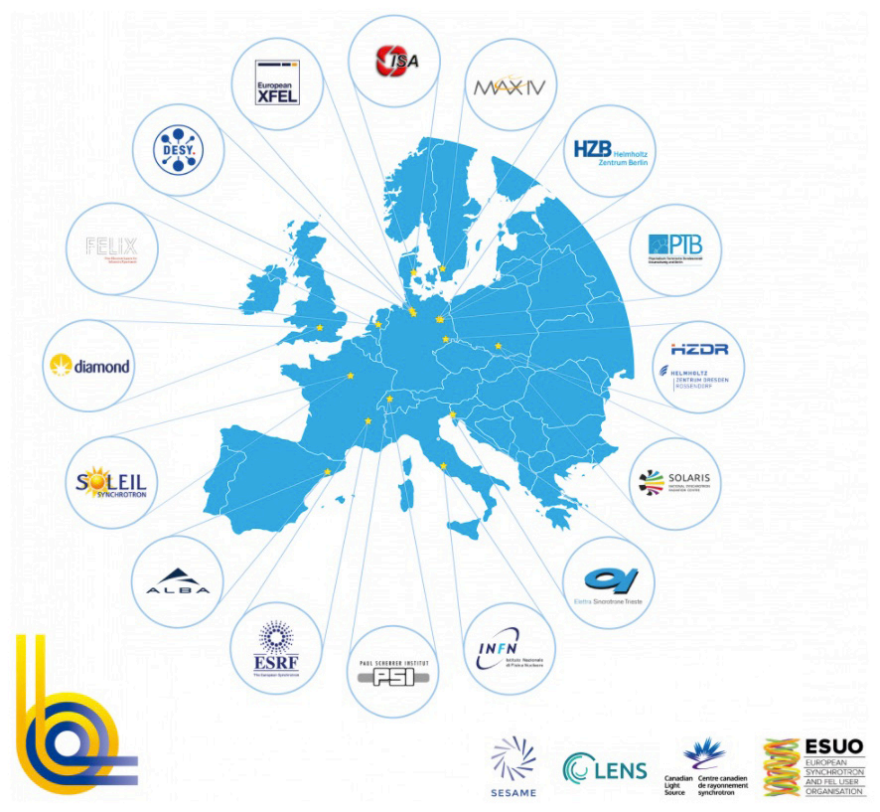
Distribution of major synchrotron facilities across Europe that are part of LEAPS (L eague of E uropean A ccelerator-based P hoton S ources).

**Figure 5.**
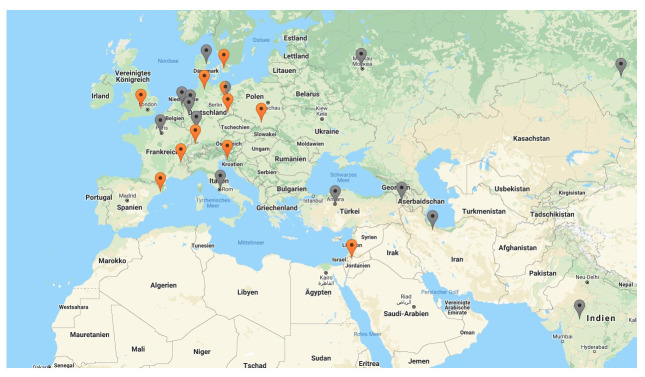
S ynchrotrons across E urope and the M iddle E ast ^16^.

**Figure 6.**
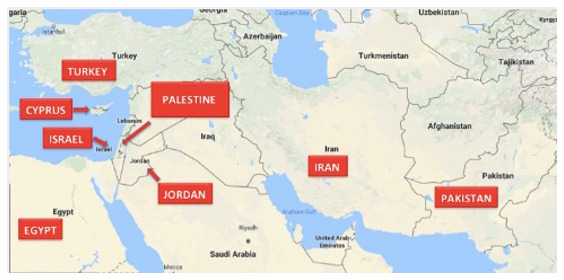
M ember countries of SESAME ^17^.

Against the background of the uneven distribution of synchrotrons worldwide ( Figure 3), the initiation and successful realization of SESAME as the first operational synchrotron in the Middle East is fairly exceptional. This is true, not only with regards to its location, yet even more so with regards to its member composition. Currently, the member countries of SESAME are Jordan, Turkey, Israel, the Palestinian Authority, Pakistan, Iran, Cyprus and Egypt, none of them possessing a synchrotron by themselves ( Figure 6).

SESAME has been modelled on the example of CERN mainly with regards to its founding ambition and political vision ^12^. CERN is the European Organization for Nuclear Research, founded in 1954 to foster trust, international cooperation, and open up room for building mutual understanding across the borders of (formerly) conflicted parties on the common ground of scientific interest and research. Technically and scientifically, there are major and evident differences between CERN and SESAME. Most prominently, CERN is geared towards fundamental research in high-energy physics and it has become the largest particle accelerator in the world, spearheading groundbreaking research and innovation. By contrast, SESAME is designed as a synchrotron *user facility* that provides technical units for a still sophisticated, yet rather standardized set of experimental applications. Regarding its purpose, SESAME is structurally better compared with synchrotron user facilities such as SOLEIL in France or Diamond Light Source in the UK ( Figure 4). However, SESAME comes only at a tiny fraction of CERN (and of the other named synchrotrons SOLEIL and Diamond) with regards to almost every structural, financial and technical aspect (number of beamlines and experimental stations, staff, resources etc.).

Just like CERN, SESAME has been founded with the vision of increasing international cooperation between scientists in a conflict-affected region ^13^. And similar to CERN, SESAME has been founded under the auspices of UNESCO, therefore being instituted as an intergovernmental organisation from the very beginning. According to the research site’s own account “SESAME will foster closer links between peoples with different traditions, political systems and beliefs, in a region where better mutual understanding is much needed.” ^14^ Additionally, the intention behind SESAME is not only to establish new links and channels of communication between Arab countries, Israel, Iran, Turkey, Cyprus, Pakistan and others on scientific grounds, but also to promote scientific excellence, education and technological development in the Middle East and therefore to represent a driving force also for the economic development in the region ^15^.

### 2.2 Research interest and methodological approach

Synchrotrons such as SESAME are captivating technologies from a scientific point of view. They provide insight into the smallest scale of the mechanisms of life that would otherwise not be perceptible to the naked eye. While the focus of this report is obviously not on the technological aspect of synchrotrons, I would like to provide a general and brief understanding of the technology in the following section to give a general idea of its relevance today ( Section 2.1). However, this report is primarily interested in SESAME as an example of science diplomacy and therefore looks at the inner workings of the international research centre with the explicit ambition of using science to further intercultural understanding. On the one hand, this case is therefore theoretically demarcated by the institutional and member structure of SESAME. On the other hand, our research interest has to go beyond these institutional limits in taking a look at the more encompassing actor network around SESAME. We look at SESAME both as an institutional structure and ask, furthermore, how it is related to (and embedded in) the global synchrotron community, and to academia and researchers in the region and in Jordan. Which actors and/or networks have been in support of SESAME and which structures have been potentially affected by SESAME, already? How do the aspirations behind SESAME act out and are tangible for researchers in the region? What is the character of the governance system and what kind of power relations can we observe? Since SESAME has been in the process of its establishment, its institutional structure has changed and evolved only recently. As part of our interest in the actors and governance structure it seems necessary to take a closer look at the evolution of SESAME in the next section, before elaborating on the current constitution and actor relations in Section 4.

We have applied a threefold methodical approach to the research for our case study within our methodological framework being oriented by grounded theory. We started with desk research, taking into account a number of lengthy and rich accounts on SESAME by involved parties. These publications mainly cover the evolution of SESAME. They are provided by former directors of the SESAME council and therefore contain insights from an invaluable first-hand experience. However, those publications are few in number, while there is a growing number of articles that take an interest in SESAME as an example of science diplomacy. They usually take a journalistic perspective, while there is almost no academic research being published about the research site itself.

As a second line of research, we generated our own data through semi-structured interviews ^18^ and conversations with participants of a users meeting in Amman, Jordan. For the purpose of interviewee selection, we assembled an overview of relevant actors and stakeholder groups and identified four main groups of stakeholders: (A) Members of SESAME Council, delegates and staff at umbrella institutions and Jordan authorities (UNESCO, EU DG R&I, members of the Jordan Royal Family), (B) SESAME local staff and directorate (Acting Director-General, Technical Director, Administrative Director, Scientific Director), (C) Members of Advisory Committees and experts at supporting research facilities providing expertise and host SESAME researchers for research visits (ITER etc.), (D) The group of users and participants in SESAME trainings.

We selected and approached key responsible actors in each group (senior officials, researchers with a long experience at SESAME) via e-mail or in person at the users meeting 2018. We designed our sample making sure to include at least one representative/perspective from each stakeholder group (purposive sampling). Five in-depth interviews with a duration between 1 and 2 hours have been conducted, four of them audio recorded, another could not be recorded for technical reasons but has been saved in written note. Written consent was obtained before the interviews ^19^. Together with the conversations that we had with participants at the users meeting our sample included council members and committee members, the current president, the director and members of the directorate level, engineers and beamline responsible persons, scientific users, administration staff and one of the founders of SESAME. As a result, our sample includes all stakeholder groups that have been identified as relevant. Furthermore, the sample is diverse not only with respect to the functions and positions of participants, but also with respect to countries of origin of the participants reflecting the international setup of SESAME.

Thirdly, as part of our data acquisition and in order to gain a personal impression, we have visited the facility and we have attended one of the yearly users meetings in Amman, Jordan (December 2018). This visit was documented with field notes. Furthermore, we visited the council meeting in December 2018 at the premises of UNESCO in Paris and had the chance to talk to a number of council representatives (member states and observers’ status).

Our approach of data analysis was open, inductive and not primarily hypothesis driven, while we had identified and developed an awareness for possible friction points throughout the preparation phase. The coding process was mainly conducted by the corresponding author and pre-structured by the analytical framework that was developed for all case studies within the S4D4C consortium prior to the selection of case studies. The analytical framework is structured as follows:

(a) A1. Governance arrangement: the formal organization of the case topic. This includes legal frameworks, rules, policy instruments, governmental strategies, official guidelines and prescribed actors. Furthermore, governance arrangements deal with the direction of implementation – i.e. top-down or bottom-up – and the structure of the arrangement, i.e. whether it resembles a hierarchical structure (where there is a clear mandate from an authority), a network structure (where processes take place in the framework of a sort of ‘epistemic community’) or rather a market structure (where supply/demand of information/action comes from different and sporadic actors and emergent needs/opportunities).
(b) A2. Stakeholder landscape: the actors involved in the case topic and their attributes (i.e. interests, roles, power to influence/facilitate/block, etc.). We are also interested in how in terms of substance and procedures and through which channels/interfaces actors relate to each other, and in the challenges of interactions between different stakeholders.
(c) A3. De facto governance practices: the de facto workings of the case topic. This involves the actual mix of all formal processes and procedures and those where actors deviate from the formal governance arrangement and perform with bureaucratic discretion. Here, it is also interesting how synergetic links are made to other social or political problem fields to further actors’ interests.

We were able to check some of our preliminary results and early hypothesis in conversations with different stakeholders from SESAME, e.g. during a presentation and poster session on SESAME at a networking meeting. 

### 2.3 Ethics

The research for this article involved human participants as volunteers for social science research (through interviews). The research, in particular data collection with human subjects, has complied with the ethical standards, policies and guidelines of Horizon 2020, which have been rigorously applied. This research has not involved any severe intervention on humans. All research was conducted ensuring respect for the participants and their dignity, protecting their values, rights and interests and fair distribution of research benefits and burden. The chosen sampling was done in a way to not systematically exclude gender, political, social and cultural views and minorities from marginal backgrounds. Participation was on a voluntary basis. Written informed consent was obtained from research participants.

## 3. History and evolution

SESAME is a unique example of science diplomacy, particularly with regards to its evolution. We distinguish three phases in the evolution of SESAME:

1. the identification phase (general promotion of vision and search for support),

2. the institutionalization phase or interim phase (initiation of the institutionalization process; inauguration of the Council of SESAME; search for structural support), and

3. the maturity phase (physical realization of the research site; construction of building, installation of beamlines, the running of a synchrotron user facility).

We have based our evidence mainly on the in-depth accounts of those who have been in charge of its establishment and operation. Herwig Schopper is former Director General of CERN as well as DESY (German Electron Synchrotron) and was an integral part of the project. He provided a comprehensive description of SESAME’s history ^20^. Furthermore, Chris Llewllyn Smith, following Herwig Schopper as president of the SESAME Council, has similarly provided a report on the history, ambitions and the challenges in SESAME’s past ^21^. We have enriched this data basis on the evolution of SESAME with interviews that we conducted with staff and stakeholders.

### 3.1 1 ^st^ phase (1980s-1999): identification phase

The evolution of SESAME can be regarded as the product of two different initiatives that have coincided ^22^. On the one hand, the idea for a synchrotron light source in the Middle East dates back to the 1980s ^23^, when several countries started to develop an interest in designing and building national synchrotrons (Interview#3). The Pakistani physicist and Nobel Prize winner Abdus Salam first promoted SESAME. Salam was an advocate for the idea of international collaboration, science and technology transfer and a general enhancement of scientific efforts. He “proposed the creation of an ‘Arabian Gulf University’ at Jeddah in Saudi Arabia, which included a synchrotron light source as part of the plan” ^24^. But initiatives to design (national) synchrotrons e.g. in Saudi-Arabia or Bahrain did not take hold.

On the other hand, SESAME also traces back to the initiative of a few distinguished high-energy physicians from the US and Germany, namely and most importantly Herman Winick (Stanford Linear Accelerator Center [SLAC] ^25^) and Gustaf Adolf Voss (former director of DESY ^26^), who were both members of the BESSY I ^27^ (German synchrotron in West Berlin) advisory committee in 1997. As such they learnt about the plan to shut down the facility in the 1990s in the wake of German reunification ^28^. BESSY I was to be decommissioned due to the establishment of a more powerful one at a different location in Berlin-Adlershof, in what was previously East Berlin. Since it would have been too costly to maintain both facilities and since the old facility would therefore not be used anymore, Winick and Voss made the case for recycling the Berlin synchrotron BESSY I. The relevant parts of BESSY I were the 0.8 GeV second-generation storage ring and injector system ^29^. It would have been the first time ever that a synchrotron was recycled. However, initially it was not intended to ship it to the Middle East. There were plans to send it to Eastern Europe, to Poland or Romania (Interview#1 and Interview#4).

Winick and Voss revived Salam’s vision ^30^ and promoted the idea to donate parts of BESSY I to the Middle East. But the first approach towards the Middle East and North Africa (MENA) region did not result in any interest in the project ^31^. This is where another important group joined the process. Only two years earlier the committee for Middle Eastern Scientific Collaboration (MESC) was founded, consisting of scientists from CERN who wanted to embrace Arab-Israeli collaboration. Initiators were the physicists Sergio Fubini and Eliezer Rabinovich ^32^. It was at a MESC seminar in November 1997 in Turin (Italy) where Voss’ and Winick’s idea aroused great interest among the 31 scientists from Israel and the Arab States that were present. A steering group was established in order to organize the work, chaired by Herwig Schopper who had just retired as Director General of CERN.

At a meeting of this group in the following year, more concrete plans were made and it was decided to reach out to potential international partners. After an informal confirmation that BESSY I was to be decommissioned and a first positive response to the idea of donation to the Middle East, Schopper and Fubini reached out to UNESCO. Federico Mayor, Director General of UNESCO at that time, confirmed his support. This was an important cornerstone since, considering the troubles of the region, UNESCO seemed to be the only way of running such a project. From the beginning SESAME was thought of as following the CERN model, also promoting science while at the same time bringing people from different nations together and serving as a peacemaking project ^33^.

### 3.2 2nd phase (1999–2008): institutionalization phase

Mayor’s support led to the first consultative meeting of MENA and Mediterranean governments at UNESCO Paris in June 1999, where the project was much appreciated and as a result the interim council was established. The function of the interim council was the development of a proposal for the establishment of the organization that could be submitted to UNESCO ^34^. In the beginning, it consisted of 12 members ^35^ and four advising committees (technical, scientific, training, finance) ^36^. Between 1999 and 2001 the interim council held nine meetings ^37^ until it was transformed into the SESAME Council in 2003. The major task in this phase was to find an appropriate location and host country, to organize the shipping and to prepare the technical design.

Schopper describes the procedure of finding a location for the research facility comprehensively ^38^. Of the 12 sites offered by seven SESAME members (Armenia, Egypt, Iran, Jordan, Oman, Palestine and Turkey) ^39^ none was really suitable. Many different kinds of obstacles got into the way, e.g. financial problems of the Palestinian National Authority, structural conditions of an Armenian building or Iran’s entry requirements ^40^. Eventually, there was a decision for Jordan in a competition between five remaining countries, among them Egypt. “Jordan was the most promising country as far as free access by all scientists was regarded” ^41^. It seems that it is more of a lucky coincidence that SESAME could finally win over Jordan as its physical home, since there was no contact with the Jordanian government, which was a large setback according to Schopper. Schopper reports that he had asked a former student and friend of his, Isa Khubeis, in his role as Vice-President of Al-Balqa Applied University at Allan for help, who invited him for dinner. Surprisingly HRH Prince Ghazi Bin Muhammad showed up at the dinner. He led the Governing Board of the same university and was advisor to HM King Abdullah II. This is how Schopper got his chance to present the project to the King. He was able to convince him of the idea of SESAME and received a written confirmation of the King’s commitment ^42^. At an interim council meeting in April 2000 the site in Allan, offered by Jordan, was officially chosen and confirmed as the location of the facility.

As Schopper reports in his article, the whole project was at risk when the German Federal Ministry of Education and Research wanted the components to be dismantled until the end of 1999. It was only by the generous financial support of Kiōchirō Matsuura, who had just recently followed Mayor as Director General of UNESCO, that SESAME’s story did not end there. Giving USD 400,000 he provided two thirds of the needed money, which he took from a sum given by the Japanese government to his own disposal. Another $200,000 donation was made available by Russia and Sweden. Thus, the components could be shipped to Jordan in June 2002 where they were stored until the construction of the building was ready for them to be installed ^43^. The building itself was decided to be a recreation of ANKA (Angströmquelle Karlsruhe), a synchrotron light source facility in Karlsruhe (Germany) in order to shorten the needed time and to save the effort of conceptualizing it from scratch ^44^.

Furthermore, a “white book” was presented in April 2002 proposing an energy increase to 2 GeV and a circumference of 120m. These enormous changes to the initial design required a larger building, which led to the duplication of the ANKA building that was mentioned before. In order to compensate for the rising costs of such changes to the original plans, new ways of financing were established. While trying to get some components as gifts from other facilities, the European Union was asked to finance the main ring. The EU was prepared to fund the ring only insofar as the electron energy would be further increased so that SESAME could keep pace with other newly built synchrotron facilities worldwide. As a consequence, a “yellow book” was set up that took into account these new requirements ^45^. Finally in 2004, the decision was made to build “a completely new 2.5 GeV main storage ring, with straight sections that can accommodate insertion devices [...], thereby making SESAME a competitive third-generation light source, while retaining the BESSY I microtron and booster synchrotron, which provide the first two stages of acceleration” ^46^.

The SESAME research center formally came into existence in 2004. Prior to that, UNESCO’s Executive Board officially had to accept the proposed statutes that had been prepared by the interim council in May 2002 ^47^. According to UNESCO rules at least six governments had to accept the statutes and join the council for SESAME to be formally founded. On January 6 2003 Matsuura could announce that this requirement had been fulfilled. Bahrain, Egypt, Israel, Jordan, Pakistan, Palestine and Turkey had become the founding members of SESAME ^48^. On the same day, His Majesty King Abdullah of Jordan laid the cornerstone of the building ^49^ and the SESAME Council held its first meeting, thereby superseding the interim council. Herwig Schopper was elected as President of the permanent council with Khaled Toukan (Jordan) and Dinçer Ülkü (Turkey) as Vice-Presidents.

Even though SESAME has achieved the formal status of an international research center under the auspices of UNESCO, it still seems to owe its formation and maybe even its continuance to the exceptional dedication of a number of individuals, generally scientists by training. This makes SESAME a prominent example for a “bottom-up” science diplomacy case. SESAME is clearly the result of strategic thinking and a good deal of sensitivity for timing and political circumstance. But most importantly, the compassion, commitment, and determination of a number of individuals, mainly scientists in high ranking or directing positions within the science system were the essential drivers at the early stages of the project.

### 3.3 3rd phase (2008–2017): maturity phase

The official opening of the building was in November 2008. The first successful electron beam was produced on 14 July 2009 ^50^. At the same time Chris Llewellyn Smith, another former Director General of CERN, took over the presidency of the SESAME Council. Under his lead a strategic plan was set up to structure the work to come and install the equipment in the so far empty building, as described in a recent article of his ^51^. The plan “revealed that […] construction would cost much more than previously assumed, and it became clear that it would not be possible to obtain all the funding from outside without first obtaining a substantial part from the Members” ^52^. In 2012 the four member countries Iran, Israel, Jordan, and Turkey each contributed USD 5 million to the capital budget. Following this, the EU funded CERN with the same sum to construct the magnet system for SESAME. Italy also gave EURO 3.35 million in total since 2014 ^53^. Following these contributions the installation started and was finished in November 2017. During that time, in 2014, the roof collapsed due to heavy snowfall but fortunately the shielding wall inside the hall protected the machine. One year later the damage was repaired without further impairments. On 16 May 2017, His Majesty King Abdullah II of Jordan officially opened the SESAME research facility “in the presence of the Directors General of CERN, the International Atomic Energy Agency (IAEA) and UNESCO, the European Commissioner for Research, Science and Innovation, senior representatives of the SESAME Members and Observers” ^54^.

Within recent years, SESAME has succeeded in operating its first two beamlines (one in the infrared and the other one in the X-ray spectrum; IR and XFAS) and in opening up the research facility to the community of scientists on a regular basis. This year marks the first scientific publication that results from research conducted at SESAME. Yet, SESAME’s future as a fully-fledged synchrotron laboratory with a number of more beamlines is still to come. The building allows for up to 18 more beamlines besides the currently installed two ^55^. Two more beamlines are currently under construction; and two more are being designed at the moment ^56^. The two currently being designed are the BEATS (BEAmline for Tomography at SESAME) beamline, as well as a soft X-ray beamline.

A facility consists not only of well-engineered technical units but, just as importantly, it requires the development of a user community (researchers) and the administrative routines in dealing with them. In support of that, the surrounding (research) infrastructure has to be developed, including spaces where scientists can gather and exchange. This includes building a guest house (soon to be finished) and a conference venue where researchers can stay during their experiments, work and meet each other. A conference venue may also be an important asset to attract the wider research community, apart from conducting research at the site. In addition to that, there was an initiative to collect money for building a cafeteria in 2015, which has not yet been realized. SESAME started public fundraising referring to the important role “played by the CERN cafeteria during the Cold War where European, American and Russian scientists could meet and messages were conveyed to governments thus bringing important results”. Finally and most crucially, SESAME is putting effort into promoting the relevance of synchrotron technology for various disciplines and research in the region as well as to support the development of competence in order to exploit the facility’s research potential, e.g. through training and users meetings ^57^.

In general, the idea of SESAME is not the result of a plan or strategy by political actors. Neither was the project born at a singular instance or place, from where it was brought into being on a straight-line roadmap. The evolution of SESAME, both as a vision and in its current institutional and physical realization, is an ongoing contingent process that could have failed, faded out or taken different tracks at several junctures. Its history is described most appropriately as an encounter of circumstance, creativity, and coincidence. SESAME depended on the right people who met at the right places during the right times: the occasion of German re-unification propelled the plan to build a new synchrotron that would replace an old synchrotron that could be “refurbished” for the first time ^58^; a few international, renowned and synchrotron-experienced scientists that had both the intuition that a synchrotron was missing in the Middle East and were acquainted with the proceedings in Germany; and finally the supportive network and example of CERN that could provide a role model, to name but the most crucial conditions. SESAME stems from a “bottom up” initiative by scientists. It was only later that the idea of SESAME resulted in an institutional framework and could rely on a more formalized structure and support on the international and national level.

## 4. Stakeholder landscape and character of relationships

### 4.1 Institutional structure

**Figure 7.**
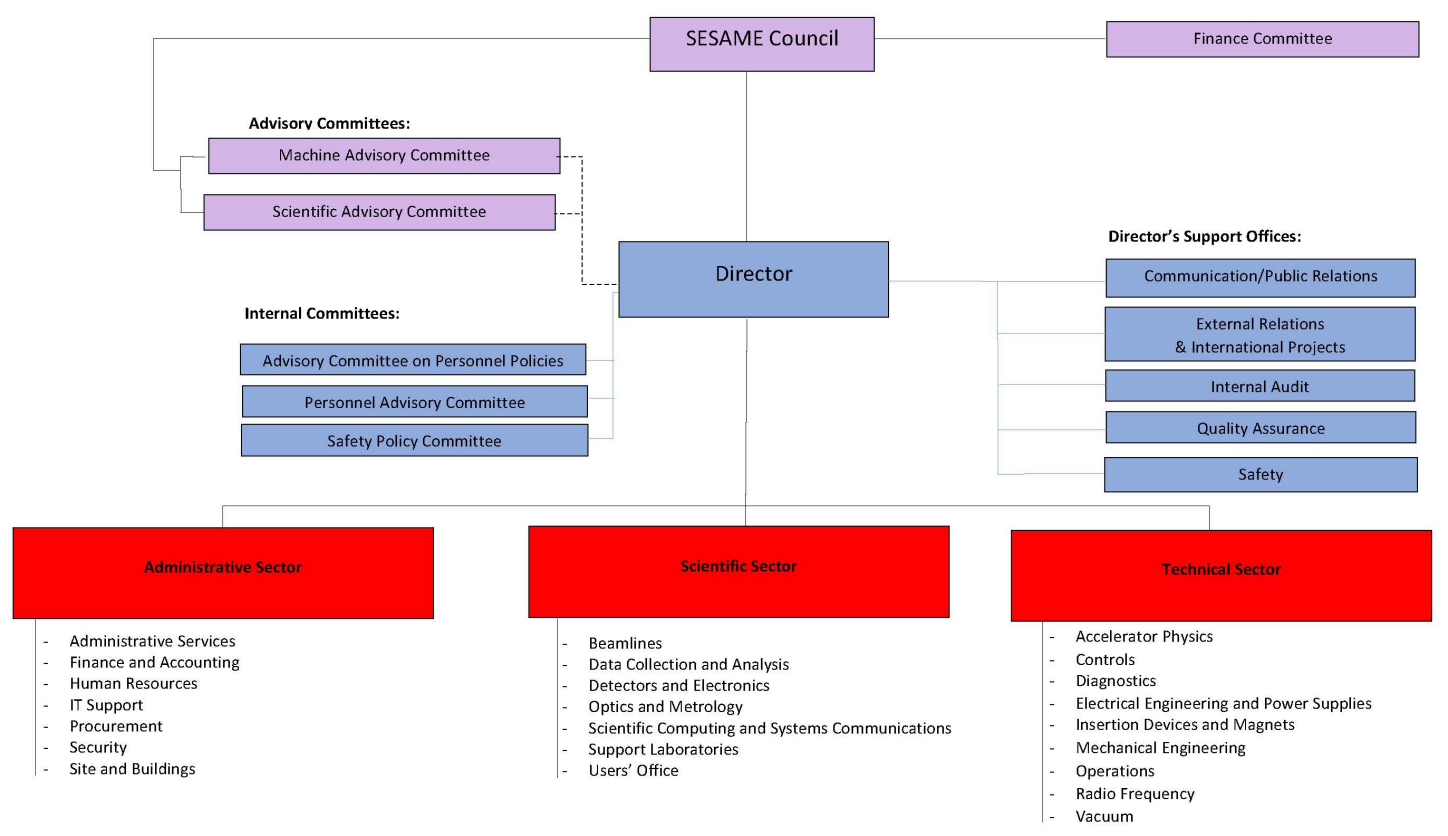
O rganizational C hart of SESAME as of 2019 ^59^.

SESAME is an intergovernmental scientific centre owned by its members and resting under the auspices of UNESCO. From an institutional point of view, this constitutes SESAME as an independent and self-responsible international organization. UNESCO serves as the legal depository of the statutes of SESAME. SESAME’s core governing body is the SESAME Council (subsequently referred to as “the council”), which came into existence on April 15 ^th^, 2004 and holds regular meetings twice a year with the representatives of the member countries and observer countries/observer institutions. Countries that are “members of SESAME” are in fact members of the council. The executive bodies of SESAME are the directorate and the administrative, scientific, and technical offices. The advisory committees are formally established at the council as well. The advisory committees constitute important operational links between the Council and the development of the facility and the running of research activities on the ground ( Figure 7).

### 4.2 Composition of the SESAME Council (member and observer states)

**Figure 8.**
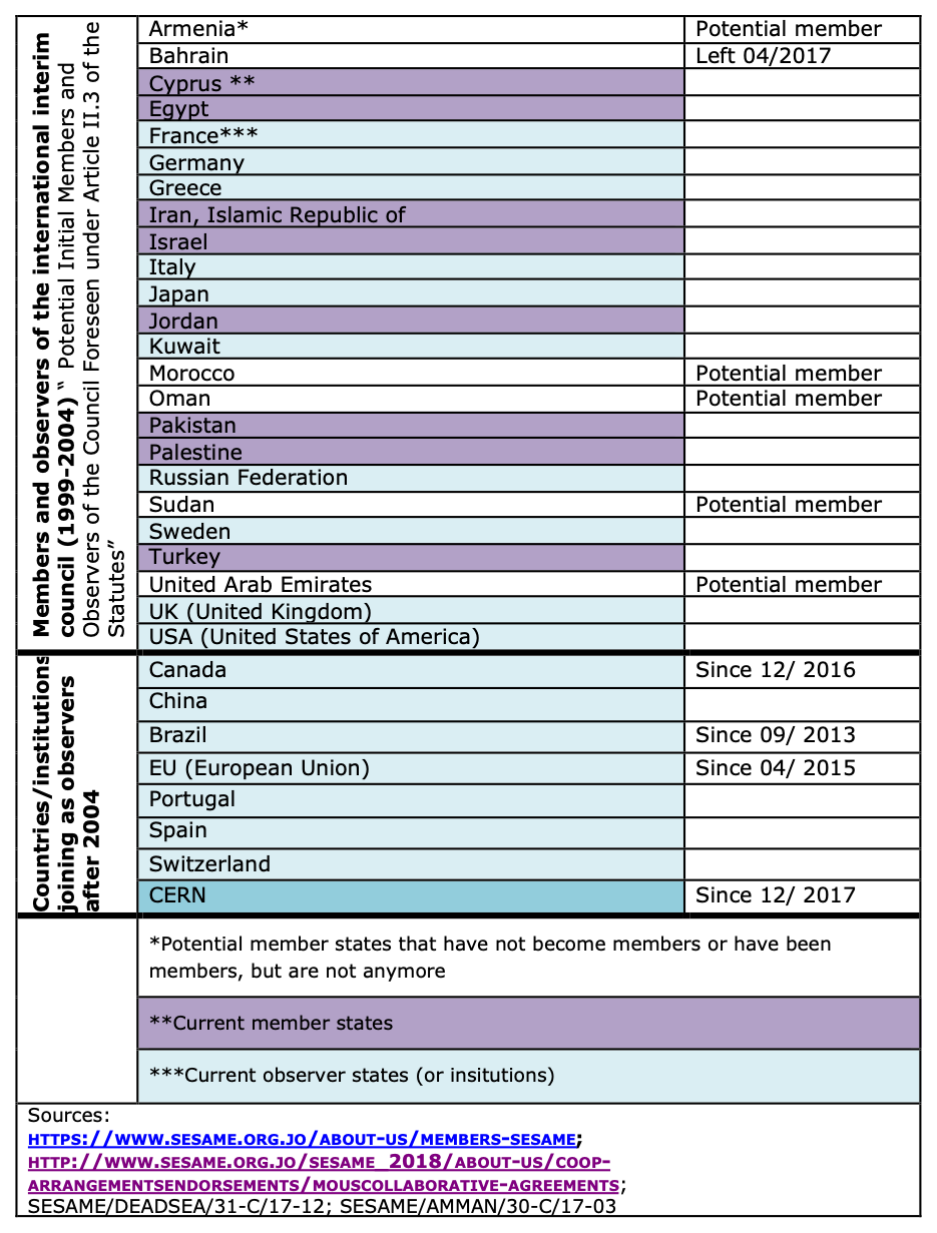
SESAME’s members and observers (own graphic).

There are two different forms of institutional membership (in the council): members and observers. The status of membership of SESAME has been awarded to countries only, while countries and intergovernmental organizations have obtained observer status. Currently, SESAME consists of eight full member states (as of 2019). These are: Cyprus, Egypt, Iran, Israel, Jordan, Pakistan, the Palestinian Authority and Turkey. Furthermore, SESAME enjoys the support of a number of observer countries; these are (as of 2017) Brazil, Canada, China (People’s Republic of), France, Germany, Greece, Italy, Japan, Kuwait, Portugal, Russian Federation, Spain, Sweden, Switzerland, the United Kingdom, and the United States of America. Also, the European Organization for Nuclear Research (CERN) and the European Union (EU) also obtained observer status ( Figure 8).

This division of membership between members and observers allows for an institutional setting, in which countries that are intended to actively participate in SESAME i.e. to use the research facility (target countries) obtain different rights and obligations than those supporting the centre. The most important formal obligation of active member countries in contrast to supporting countries or supporting institutions is the yearly financial contribution, while observers have no formal obligations. Given the fact that UNESCO is an intergovernmental body and given the statutes of SESAME it can be concluded that the only major sanctioning mechanism of “non-compliant” members consists in the eventual exclusion from the council and revocation of membership.

We conclude that countries that choose to be active members of SESAME have been able to stipulate sufficient “intrinsic” motivation within their countries, i.e. identified the associated national interest of their membership in SESAME or indicate a strong and convincing proponent in the national research infrastructure that is able to encourage the obligatory authorities to commit the required budget and political will. This also holds for the observer states, e.g. the German Ministry for Education and Science was described as very hesitant to support the facility project from the very beginning. It never stepped in due to its assessment that the project would be too risky and at one point even wanted to stop the export of equipment to Jordan ^60^. By contrast, German science organizations acting independently from the government have been the main driving force behind the support and membership in the council of Germany.

SESAME has been trying to reach out to and affiliate more member countries, especially from the Maghreb and the Gulf ^61^. For instance, there were concrete negotiations with Iraq and interest for the country to join in November 2013, yet Iraq has not become a member due to discord about its required yearly financial contribution ^62^. Furthermore, Armenia, Morocco, Oman and the United Arab Emirates were initially part of the interim council, but did not make the transition into the current formal membership (since 2004) for both financial and political reasons. Since 2004, membership is tied to the yearly contribution. Bahrain was one of the formal founding member countries, but is not a member anymore. Bahrain’s membership ceased in April 2017 after the country had not contributed its financial share since 2005 ^63^. This means that the country has most likely only contributed its initial first year share. Before Bahrain’s exclusion, the country had been formally asked to re-engage with SESAME by the council. Saudi Arabia is not part of SESAME due to Iran’s membership. This might be one of the reasons why the United States are not engaging more strongly in SESAME on the governmental level.

### 4.3 Agency and representation in the council

As far as it has been revealed to us, none of the representatives in the council (therefore, also not the president of the council) receive any salary on behalf of SESAME. They contribute in their positions on a non-stipendiary basis or as part of their official affiliation such as the liaison officer of UNESCO, Clarissa Formosa-Gauci. Representatives of member countries and observers as well as members of the advisory committees typically participate and contribute on behalf of their main employing institutions. Representatives of the member countries usually work either in high-ranking positions as professors and directors at universities, academies or synchrotron facilities in their country or in the respective ministries for education, research and innovation. A considerable share of delegates are furthermore closely affiliated with national atomic energy agencies in their respective countries. Only a few countries with observer status are represented by an official envoy or the ambassador to the UNESCO (diplomatic corps) in the council. We have found that it speaks for the authentic engagement of a country if the representative is participating on a regular and long-term basis (is not replaced often), takes a personal interest in the project and is affiliated with a scientific institution in his or her home country. We found that there was a considerable personal engagement and concern with advancing matters at SESAME from representatives that were sent by research institutions. However, the council meetings are not open to the public and therefore insights were restricted also to us.

It cannot be overstated that the outlook of SESAME depends to a great extent on the various institutional affiliations and the scientific expertise of individuals, who serve in any of the permanent and honorary positions in the institutional setting of SESAME, mainly in the council and the directorate. They bring in their professional international networks and expertise and in that have made possible the establishment of an international research infrastructure from scratch. Most importantly, this certainly applies to the former and current presidents of the council that have also served as former directors of CERN. They have contributed and still contribute an invaluable asset of experience, expertise and network (we will take a closer look at them in the next sections).

### 4.4 Advisory Committees

The advisory committees are formally a part of the Council. They have played (and still play) a significant role in the planning, technical support and active international promotion of SESAME throughout its establishment. Most importantly, they seem to form crucial junctures to feed in external technical expertise from the international synchrotron light source community. In particular, the Scientific Advisory Committee (SAC) and its respective chairs have advanced the makeup and outreach of SESAME considerably. Since 2018, the SAC has been chaired by Esen Ercan Alp, a senior scientist of Turkish descent and a long serving physicist at the Advanced Photon Source at the Argonne National Laboratory in the US. Prior to him, the position was filled by Professor Zehra Sayers from 2002 to 2018, a biophysicist and director of the foundations development at Sabanci University, Istanbul. Sayers has served not only with her international expertise but also with great passion and commitment that is certainly required to execute the task in the phase of institutionalization and construction of the synchrotron. She was awarded the Rammal Award 2017 for her outstanding contribution, “from collaboration at the political level and at the construction of the facility to getting the science going by ensuring high quality exciting projects of young and experienced scientists from the region”. Sayers has given several TED talks about her commitment to SESAME. In these talks it becomes evident that working with synchrotrons has touched upon her life and this is from where she draws her motivation. According to her, SESAME became ”a very big passion in my life”, ^64^ the fascination for “making the invisible visible” that she discovered also during the influential experience of working in Hamburg at DESY as a young PhD scholar (also a station in the academic life of Esen Alp). She describes the working atmosphere at a synchrotron as a very energizing and inspiring one: ”synchrotrons have a special atmosphere or environment of their own” ^65^. She articulates the urge to give back something to the next generation of scholars and to leave something behind.

Similarly, the Technical Advisory Committee (TAC) and the Training Advisory Committee (TrAC) are staffed with international personnel that dispose of considerable expertise and specialization in the field of synchrotron physics. Amor Nadji, a current member of TAC, is Director of Accelerators and Engineering at the SOLEIL synchrotron user facility close to Paris, France and Professor Javad Rahighi, currently the chair of (TrAC), is Professor of experimental physics at the Institute for Research in Fundamental Sciences in Tehran, Iran ^66^. It may have been a blessing in the constitution of SESAME that synchrotron technology is still such a fairly exquisite and specialized area of expertise so that careers in this “world” are often international and networks span globally. The TAC has worked towards building expertise to use synchrotrons in the region and has therefore organized workshops and training at other facilities in Europe and beyond.

### 4.5 Permanent staff

The permanent staff at the SESAME research site are still fairly limited, at around 45 people ^67^. They are mainly composed of technical engineers, management and staff required to run the facility and the two beamlines. Permanent staff are not to be confused with the users (researchers) that come to SESAME to conduct experiments ^68^. The permanent staff are truly international, and are recruited from the local area, from the Middle East region and from the international (synchrotron) community beyond the Middle East in accordance with SESAME’s vision. For example, the two responsible beamline scientists of the two currently operational beamlines are Messaoud Harfouche (formerly serving at the Paul Scherrer Institute in Switzerland, XAFS/XRF beamline) and Gihan Kamel from Egypt (previously having been trained in Italy and serving in Egypt; since 2000 the only woman in the permanent staff at SESAME; IR Beamline). If we decide to understand science diplomacy as the creation of bonds between people with different backgrounds on a concrete level (as a crucial aspect of the abstract aim to contribute to peace and mutual understanding), it is achieved during the daily business at SESAME. This includes the daily encounters of internationals who spend a lot of time together, sharing commitment and effort to reach common aims ^69^. This commitment was described as very strong to us. It is also based on the high individual motivation and openness to a common cause.

However, the staffing level is thin due to budget restrictions, especially due to the insecurities about the member contributions ^70^. Responsible positions are not necessarily equipped with a full-time position.. It is intended that “in the long run, the position of Secretary of the Council will move to SESAME. In the meantime, it will be held by Clarissa Formosa Gauci who has de facto been carrying out the tasks incumbent on this position ever since the creation of SESAME in 2004 and before this during part of the period of the Interim Council.” ^71^ The director of SESAME, Khaled Toukan, serves on a non-renumerative basis. Khaled Toukan is also the president of the Jordan Atomic Energy Commission and held several positions as minister for science and education in the Jordan government. This provides SESAME with invaluable backing in its host country and it shows that science diplomacy is not necessarily a matter of “in between” (in between different organizations, between scientist and diplomats), but it is maybe even just as much a matter of personal union.

### 4.6 Institutional environment and support from observer institutions (incl. the EU)

Even though SESAME owes its initiation to a small number of individual physicists, it would not exist without the on-going support of key international and supranational institutions and its incorporation into this institutional environment. The main institutions in this environment are UNESCO, CERN, IAEA and the European Union. UNESCO has lent the institutional framework, formal recognition and the official reputation as an international science diplomacy endeavour to SESAME. The IAEA and the Jordan Atomic Energy Commission are similarly important to provide scientific support and also have helped to provide necessary political backing within Jordan. CERN had a major impact on the technical evolution of the synchrotron and provided the constitution and *raison d’etre* of SESAME as a prominent and successful role model. The EU has made numerous financial contributions and enabled contributions of equipment and exchanges by experts from other institutions and member countries through its allocation mechanisms. It joined the project after the late institutionalization phase, however. Recently, SESAME has joined LEAPS (League of European Accelerator-based Photon Sources) as an associate member and in that it has also taken a step forward to the next level of affiliation for the self-governance of European synchrotrons.

CERN has not only served as a role model and provided the overall institutional setup (institutional structure, statutes) but also lent a strong and easy to grasp narrative to the project. This immaterial contribution should not be underestimated. It was invaluable for the understanding and promotion of SESAME by the international community and the public ^72^. This kind of support by CERN has contributed credibility to its mission and development and it has encouraged commitment by other stakeholders. Furthermore, and much more tangibly, it has also equipped SESAME with machinery components, technical services and expertise in the construction of the storage ring. “CERN’s contribution was hugely beneficial, and working with CERN’s experts provided wonderful training experience for SESAME staff. The voluntary support from the Members also encouraged Italy to provide EUR 1 million in 2014, which was used to procure accelerating cavities; this was followed by further Italian contributions, so far amounting to a total of EUR 3.35 million of which the most recent part is being used to build a hostel for SESAME users.” ^73^ (Smith 2018, 180)

CERN’s support for SESAME was also made possible through the European Union, which provided CERN with EUR 5 million to “lead the procurement of the magnet system for the main ring in collaboration with SESAME” ^74^. The magnets are key components of a synchrotron. They have been designed by the technical team from SESAME in the first place, while scientists from CERN provided a review and conducted the measurement of the magnets, which requires expensive equipment that SESAME does not have on its own, and also helped to set them up ^75^.

SESAME staff and stakeholders do not become tired of pointing out the many important contributions the EU has made ^76^. The EU support has been described as comprehensive and coherent ^77^. The EU has made and/or enabled numerous financial contributions more recently, mainly within the last few years. The EU provided EUR 6.36 million (own estimation upon the given number of USD 7.05 million) for the construction of an on-grid solar power plant through the Jordanian government in 2016. The power plant was officially inaugurated in February 2019 and is located 30 km away from the research facility. It will be able to satisfy SESAME’s full energy demand in the years to come: “Thanks to this power plant SESAME is now not only the first synchrotron light facility in the region, but also the world’s first large accelerator complex to be fully powered by renewable energy.” Again, the Jordanian authorities, in this case especially the support from the Jordan Atomic Energy Commission (JAEC), have been key in realizing this effort. Since the high electricity requirement has been a major concern for SESAME and has even forced a stop of the running of the beamline for weeks during the year 2018, the new power plant is a big step forward in terms of financial security and budget reliability ^78^. For the first time, the EU as a donor has invested in an effort that helps to cover the running expenses, by contrast to the majority of contributions that went into technological expansions and enhancements of the facility.

Furthermore, the EU has provided EUR 5.97 million through a Horizon 2020 grant to design and install the fifth beamline, producing hard X-ray light for tomography (BEATS), beginning in 2019 and in cooperation with ESRF (European Synchrotron Radiation Facility). The major advantage of this project and its funding scheme is that SESAME staff are in charge of conducting all the single steps of setting up the beamline. This is different in comparison to other donations and cooperation with European synchrotrons that have sent their own engineers for the set-up of the donated equipment ^79^. In addition to that, the EU has provided funds for the “OPEN SESAME” consortium consisting of 10 European synchrotron facilities and science organizations with funds to establish an orchestrated training and promotion programme that is tailored to the needs of SESAME within the Horizon 2020 framework programme ^80^. The project started on January 1, 2017 and ran for three years. It also aimed at having a “lasting impact on a reinforced European Research Area, and particularly in strengthening international cooperation for research infrastructures with a key Region located close to Europe.” Finally, the EU funds a project called “Convenient Access to Light Sources Open to Innovation, Science and to the World ( CALIPSOplus)”, which is geared towards supporting the user community and research trips to SESAME. It mainly funds research trips to SESAME for researchers coming from the member states Turkey, Israel and Cyprus ^81^.

### 4.7 The Global Synchrotron Community

The global synchrotron community is the group of scientists and engineers that design, build and maintain synchrotrons. Even though it has a global span, this community is fairly small and intimate. In general, members know each other ^82^. This is different from the synchrotron *users* community, which is far larger (it amounts to around 25,000 scientists at the European facilities alone), rapidly growing and much more diverse in disciplinary background. There is a frequent exchange of expertise and personnel between the facilities in the world; according to LEAPS founder Helmet Dosch “Synchrotron x-ray sources and free-electron lasers have always collaborated, but not in a coordinated way“. The community is competitive when it comes to designing and experimenting with more powerful and innovative technologies. Yet, it also has proven to be highly cooperative and engaging when it comes to sharing expertise and supporting each other in the construction of new synchrotrons. Furthermore, the community is still evolving and constantly (re-)organizing ^83^.

The intimate character of the synchrotron community is linked to the fact that synchrotrons are sophisticated machines that require highly specialized expertise and therefore attract and bring together a small group of people. The cohesion of the group is also due to the fact that there are few advanced synchrotron light sources around the world where people are trained and develop the technology. Furthermore, synchrotron technology is fairly young (the community has transitioned into the 2 ^nd^ generation). Three decades passed before synchrotrons could be used as the users research facilities that they are today. Until the 1980s the technology was still in its infancy and the community was accordingly smaller. It was in the early 1990s that more countries – besides the few initial sites in the US, in the Soviet Union, in Germany and in Switzerland – constructed facilities for a broader range of research applications. Consequently, the few founding figures and early developers of this technology were imperative advisers for the establishment of new synchrotrons. In the late 1980s, for example, Taiwan and South Korea decided to build synchrotrons as part of their investment strategy in innovation with the financial resources coming out of export excesses from the growing low technology industries (in clothes, toys, electronics etc.). They therefore depended on expertise outside of their country. A major motivation was to train students on an internationally competitive level and to prevent brain drain ^84^. They succeeded in training hundreds of students and in building up their own users communities in the region even with a limited performance range of their facility ^85^. Therefore, SESAME is not the first synchrotron that was built on the basis of the support of the global synchrotron community in a country that is completely new to the technology.

The biggest difference is the member constellation and structure of SESAME that again has a major impact on the financial support, especially income reliability and user structure. These countries such as Taiwan and South Korea were ready to spend USD 100 million on this technology and succeeded in building up highly active users communities that carried the synchrotrons into self-sustaining futures ^86^. Similarly, there is almost no experience with accelerator technologies in the Middle East. Usually, scientists from the region have to go to facilities in Europe or the US to be trained and to conduct research if they get a chance. It will be a major challenge for SESAME to also create the scientific awareness and demand not only within one country but a whole region to attract users.

Looking at the overall personnel structure (meaning people who have lend their expertise and time during the management, design, construction and running of the site), SESAME is brought into being by the global synchrotron community. It is based on the support of a large number of facilities in Europe and the United States that have contributed components, expertise, training and exchanges. Apart from that, the current staff at SESAME were also recruited from these synchrotrons. These physicists come from different parts of the world and usually have worked at several synchrotrons around the globe. This also includes scientists from the Middle East region that went to other countries to study and work before they now returned to the region and before getting involved with SESAME. Seen against this background, SESAME is a product of what we call the global synchrotron community and in that it is truly an international science endeavour.

## 5. Practices, interfaces and frictions

### 5.1 Training

Offering training is one of SESAME’s main activities and objectives. Before SESAME started to serve as a research facility in 2018 and before it even operated its first beamlines, it organized training at different synchrotron facilities and tried to bring academics together in order to create an interest and understanding of the technology for many years. The intention of creating a community in the region that is familiar with synchrotron technology might be just as important as building the research site itself. Given the fact that there have been only two research teams present in 2018, the main “interfaces” that are of interest from a science diplomacy perspective are still training, users meetings, and the collaboration during the designing and commissioning of the facility. Furthermore, the training is another instance of international and cross-cultural encounter and collaboration. At these events, scientists from the region and from the international synchrotron community meet each other in one place, which they would not necessarily do during the short research stays at the site.

In the case of SESAME, training mainly deals with “accelerator physics, beamlines, and scientific applications” ^87^. The purpose of the training is pursued in different settings and frameworks. It does not only include traditional workshops and individual trainings through visits at other synchrotrons and fellowships, but also includes the annual users meetings ^88^. Training opportunities at SESAME get funding and support from a large number of national scientific societies and international organizations: IAEA, UNESCO, ICTP ^89^, ESRF and a large number of national synchrotron facilities in Brazil, France, Germany, Italy, Japan, Portugal, Spain, Sweden, Switzerland, Taiwan, UK, USA ^90^ and this is another example for the importance of the global synchrotron community for the furthering of SESAME. A recent individual exchange example is the Diamond ^91^-SESAME Fellowship Grant, endowed with the amount of GBP 1.5 million for a time frame of three years (2017–2019). It funds the administration and mentoring efforts of SESAME staff at the DIAMOND site for visits of one to three months ^92^. It is required that the selected fellow is both a staff member of SESAME and has the nationality of one of the member states. The selection process is in the responsibility of SESAME management ^93^.

However, one of the major and institutionalized training efforts has been realized through the EU Horizon 2020 funded “OPEN SESAME” consortium that started in 2017 and was scheduled for three years and funded with EUR 2 million. The main objective of OPEN SESAME is to “train SESAME staff in the storage ring and beamline instrumentation technology, research techniques and administration for optimal use of a modern light source facility, to build-up human capacity in Middle East researchers and to train SESAME staff and its user community in public outreach and corporate communications”. The consortium’s core activity consists of 65 staff exchanges to the 10 other consortium member synchrotrons and science organizations in Europe and five training schools. It furthermore provides online learning materials and fellowships for students at the Master and doctoral level and a “roadshow” to promote SESAME’s scientific purpose in the region.

Apart from the training efforts tailored to the researcher community in the Middle East, SESAME trains engineers and electricians at its facility as part of the positions that operate the accelerator and storage ring. It therefore also “produces” well-trained staff in the region that are sought for by others, such as employers from the industry. Unfortunately, SESAME has already had the experience of losing well-trained staff to the industry ^94^. While this is of course a good sign for the quality and acceptance of the site, it is also a costly risk.

### 5.2 Users Meetings

The users meetings take place once a year, usually in Jordan or in another member country at a conference venue (also due to the fact that there is not yet a conference venue at the SESAME research facility). These meetings are the main encounters (“interface”) of the synchrotron research community in the region and researchers from the global synchrotron community, some of them working for SESAME (including members of the advisory committees, the Council president and the directors of SESAME). Users meetings are in general a means to provide the community of researchers (that are involved in a wide range of research applications and different topics) with a platform. The talks also provide current information on the state of affairs of the facility and of the selection processes and time schedules. In the case of SESAME, the users meetings are also employed as a means to teach and give general insights into the synchrotron technology and its potential applications. In that regard, the talks explicitly entail pedagogic ends and speakers, who are invited from other facilities mostly in Europe and the US, and are asked to incorporate this purpose in their presentations ^95^. Participation at the users meetings is restricted and requires application through the online SESAME Portal. Users are selected based on the scientific quality of their abstracts ^96^.

### 5.3 Researchers at SESAME and users access management

A researcher’s access to synchrotron user facilities is commonly restricted through a peer review selection process. Researchers (“users”) need to apply for “beam time”. This is also the case for SESAME. Researchers submit proposals and announce their research ideas in response to a call for proposal that is issued twice a year according to the schedule. ^97^ The proposals are selected on the basis of scientific merit and technical feasibility by an international board ( the Proposal Review Committee, PRC) that assigns a certain time frame for the researchers: “The PRC members are appointed by the director of SESAME after seeking suggestions from the members of SESAME’s Scientific Advisory Committee. They serve in a personal capacity, and hold office for three years. An additional appointment of three years is possible, but not automatic.” The evaluation of the proposals includes an initial safety assessment and the assessment of technical feasibility by the relevant beamline scientist. Users are strongly invited to contact the beamline scientists before submitting proposals in order to assess and adjust needs and possibilities beforehand.

The first call for the IR EMIRA-Beamline resulted in 43 proposal submissions. On the one hand, training and promotion efforts have proven to be successful: 12 proposals were submitted by scientists, who got involved through the OPEN SESAME schools. However, the facility was not ready soon enough for the created research demand. In its first year of official operation (2018), research at SESAME could be conducted only during approximately 15 weeks. The first round of calls for the IR beamline was submitted as early as 2013. The first round of calls for the XFAS BASEMA-Beamline was published in 2017 and resulted in 19 selected proposals. Yet, only one experiment could be conducted at each beamline until the end of 2018. In 2018, two research teams (from Cyprus and from Egypt) have conducted research at SESAME so far. Due to the delays in the running of the beamlines, the original schedules could not all be maintained and confirmations could not be adhered to, which has created dissonances and frustration among the users community already. One scientist reported that the interest and trust in collaborating with SESAME has been damaged due to recurring delays in the start of the operation ^98^. SESAME is certainly in a crucial moment in its evolution, specifically at this moment of transitioning into a fully operational facility while building up more beamlines on a permanent basis. This moment in time requires fortified investments and community building while academic results need time to build up and cannot prove the scientific merit of the project immediately.

### 5.4 Funding and financial situation

As a member-owned research centre, SESAME’s financial resources are to be primarily generated on the basis of member contributions. Reliable and timely payment of the member countries’ contributions has proven a major challenge from the start. The yearly contribution expected from each member country is adjusted to the country’s spending capacity. In the year 2018, Iran, Israel, Pakistan and Turkey were expected to pay USD 913,000 as each one’s yearly share. Cyprus, Jordan and Egypt were expected to pay approximately USD 520,000 each. Palestine was granted to contribute the smallest share of around USD 60,000. These yearly shares have been alike in former years. Cyprus, Israel, Turkey and Jordan seem to be able to satisfy their financial obligations overall, while other countries are in arrears. The financial situation is strained due to the persistent payment defaults of several member countries.

The reasons for payment delays are manifold and different for each country. In some cases, a lack of governmental support may contribute to the low payment morale ^99^. For example, the Egyptian contribution was frozen during the Egyptian revolution 2011 and regime change. In other cases, payments could not be transferred due to international sanctions despite the willingness of the member. This has happened in the case of Iran. In general, the member countries commonly take their contribution from the respective science and research budgets. Yet, most member countries allocate a comparably small share of the national budget to the science and research budgets ^100^. The Council’s leverage to sanction payment defaults is limited to the eventual exclusion of a member. (This has happened only once so far in the case of Bahrain.) Yet, from a rational choice point of view, the exclusion of a member further reduces the potential source of income for SESAME and therefore it is unlikely to be in the primary interest of the Council. This is especially true during the construction and extension phase, when initial investment requirements are high.

The two main expenditures in terms of running costs are staff and electricity. Electricity consumption amounts to approximately USD 1 million per year, an equivalent of up to 30–40% of the current annual budget ^101^. This is also due to the fact that electricity prices in Jordan are high. Jordan does not possess its own oil springs. Most of SESAME’s electricity consumption goes into the magnets for the acceleration of particles. The required basic energy input to build up the energy level in the storage ring is fairly the same for one, two or more beamlines. From a point of view of efficiency it would be therefore crucial to complete further beamlines as soon as possible. However, the budget based on the yearly contribution of the member shares does not cover more than the running expenses (approximately USD 3,000,000 in the year 2018). This means that SESAME depends on additional (perhaps external) contributions when it comes to initial investments, extending the facility or adding new beamlines.

### 5.5 Ownership and support

External financial support and equipment donations could be secured during all phases of the construction of SESAME for individual purposes and were crucial for its constitution from the very beginning. Additional national resources have been tapped in several instances on an almost regular basis to collect the instruments and expertise in order to set-up the first beamlines: some member countries have contributed extra shares; countries, science organizations and institutions with observer status have contributed financial resources, e.g. Germany, Japan, Sweden, Italy, Russia. But this was not on a regular basis or in disproportionate amounts. Despite the great support from Jordan it seems crucial however that SESAME is not perceived as a national project, neither as a project of Jordan nor any other member country among the members and users. The Jordanian Royal Family representing the country's national support for the facility was described as very supportive. Yet, their ability to support the facility was described also as limited due to the fact that the facility should not be perceived as a national project ^102^. This is true for any other funding and supporting partner from inside or outside SESAME, so that it maintains its character as an international research site that is run equally by all its members. Against this background, it seems to be important that contributions can be made through international or supranational bodies and scientific institutions. This is also an important asset of the EU as a partner of SESAME.

On the one hand, it plays an important part for the character and coherence of SESAME as a self-governing research centre (and therefore exceeds the financial aspect) that its member countries are able to gather sufficient support within their own countries to sustain the facility ^103^. On the other hand, it can be deemed unfortunate that they are not allowed to do so. Considering that the overall required budget is comparatively small for a synchrotron of this size and in regard of the overall honourable ambition of the project, there are individual donors within the country and beyond that could easily increase their share and even would be willing to do so ^104^. In comparison to the construction of national synchrotrons, SESAME seems to have also suffered from being somewhat “doomed” to be set up as an international research centre with the explicit expectations that countries have to cooperate and contribute equally. It remains a hypothetical question, if it might have been easier and faster to finish the synchrotron in smaller membership constellations.

The dependence on external contributions from other countries, supranational organizations and scientific institutions entails risks and frictions ^105^. First, it has prolonged the construction considerably. During its reconstruction, BESSY I had to receive a number of technical upgrades. Among others, it was necessary to build a new storage ring to make SESAME a competitive machine, therefore not using the BESSY I storage ring as initially intended; ”In December 2004, the design of the SESAME machine for a final energy of 2.5 GeV was approved – this meant building a new storage ring from scratch with a much larger circumference (133.2m) rather than upgrading that of BESSY I.” Many components have been added also by contributions from other synchrotrons and supporting facilities (e.g. from the UK and France ^106^), which again took time to organize, ship, and attach.

Furthermore, the dependence on donations and contributions apart from member contributions entailed the risk of misinterpretations and of creating the image of a donor-receiver asymmetry ^107^. In fact, BESSY I was an outdated facility and had been in use for decades when the decision was made that it should be replaced and decommissioned ^108^. Despite this, the idea was brilliant to decommission and recycle a synchrotron with the intention to “sell” it as a starting point for the construction of a new synchrotron. It would have been much harder (if not impossible) to create sufficient support to start a synchrotron project from scratch without any initial assets. Therefore the donation of BESSY I can be considered first and foremost a successful micro science policy strategy to convince official stakeholders and to create the founding narrative that might be strong enough to carry the idea into being. Yet it also created the impression among scientists in the region that outdated equipment was donated that was not of use in Germany anymore ^109^.

### 5.6 Science diplomacy, international collaboration and scientific excellence

This impression was even aggravated with SESAME being linked to a political vision of “bringing peace to the region” and with being established as a science diplomacy project ^110^, a narrative that is potentially imbued with a number of non-scientific intentions that are hard to read and guess for all stakeholders in the field. In general it can be stated that the political imprint of the project has not only pushed SESAME forward with regards to raising support and commitment, but it has also raised further suspicion mainly among scientists from the region. The narrative of science diplomacy as being part of SESAME’s mission was interpreted as such a political agenda that was rejected for this science collaboration effort. Some scientists were eager to point out that the project will be successful only on scientific grounds and that political motives are potentially damaging for its further success ^111^. It seemed as if the support of political stakeholders (both from a national and supranational level) was regarded as charged with intransparent motives that were potentially hampering scientific goals or were even interpreted as a disguise to impose political goals ^112^. The explicit aim of bringing people together was described by some people as a political agenda and was sometimes assessed critically or as a somewhat artificial obligation ^113^. The fact that SESAME and the support of the EU would not have been realized without Israel being a member of SESAME (a condition that is unlikely to apply to any other member) plays a part in its perception and in caveats and insecurities about the political agenda ^114^.

When it comes to the science diplomacy intention of SESAME, to bring people from conflicting national and cultural backgrounds together, assessments and results cut both ways: on the one extreme are scientists that clearly oppose working together in (what they find to be) imposed teams consisting, for example, of Israelis and Palestinians. This does not mean that they oppose SESAME as the scientific effort but rather seems to resent the idealistic charges and expectations of SESAME that from their point of view clashes with reality. One scientist said he could not ignore what Israelis have done to Arab communities and directly linked his disapproval of working closely together with Israelis (apart from taking notice of published results) to the political situation: “If an Israeli comes to SESAME he has the right to work. I do not care. But collaborate with him: No. Frankly speaking. All people in the region will do the same.” ^115^ Furthermore, he pointed out that a group of scientists left the room when an Israeli scientist presented during a recent users meeting for the same reason ^116^. He added: “We cannot accept the fact that there were things done wrong against our society, our people.[...] This dream is not coming true. It is not coming true. Unless the Israelis understand that this has to change, the owners of the land should go back to their homes, the refugees should go back to their towns. We are not against Jewish people.” ^117^

At the same time and on the other hand, SESAME is praised by scientists, who work at SESAME, for its vision and endeavors to bring people together beyond conflicting boundaries on the grounds of the uniting capacity of science. It is described as an “oasis of advanced science and technology, of understanding, neutrality, and fairness. An oasis of peace; a certain kind of peace that calls no diplomatic deals. Just science. The very pure logic of science.” ^118^ While this assessment is much more positive and affirmative with regards to the peace building effects of SESAME, it should be noticed that again the scientific nature of SESAME is emphasized beyond all others and is particularly juxtaposed in opposition to diplomatic ends: “I don’t think that the governments of SESAME Members or Observers are wasting their time or money for nothing. There are politicians, policymakers, diplomats, administrators, but the key players are scientists, engineers, and technicians. The end product is scientific results, not agreements, regulations or measures.” ^119^ Regardless of how much one is in favor of the peace making aspect of SESAME, it seems to be consensus that it will only materialize *as a result* of the scientific achievements and progress of SESAME. It will not be the result of a concrete strategy or immediate aspirations that may as well run under the heading of “science diplomacy”.

It can be summarized that scientific international collaboration at SESAME will only be successful in the sense of being inclusive and coherent towards the region if the scientific aspect is prioritized above all others ^120^. The directors, scientists and managers that are involved in SESAME and that have talked to us did not get tired of highlighting the importance of advancing SESAME in terms of scientific excellence. If SESAME does not prove that it is able to produce competitive scientific results, it will also not be able to serve in diplomatic terms. SESAME will not be a credible science diplomacy case if the scientific ambition is not considered paramount.

### 5.7 Inclusion and exclusion of actors

The topic of participation and inclusion/exclusion is a central and vigorous one in the case of SESAME and plays a role on at least two different levels. On both levels the relevance of this topic is “real” in the sense that it can be empirically substantiated; yet it clearly depends on how one chooses to look at the case. On the first and probably more obvious layer, the relevance of the topic of participation is the case in as much inclusion is regarded as a constitutive part of the founding narrative and publicly transmitted identity of SESAME. This addresses the core idea of this project, which rests on the vision of bringing together different people from different member countries on a scientific basis that do not usually cooperate. On this layer, inclusion and exclusion as a topic takes place mainly between the member countries according to differences or even hostilities between them that feeds into the founding myth of SESAME. However, this perspective relates only to the rather obvious layer of the topic of participation in this case.

On a second and more tacit layer, the topic of inclusion and exclusion may play another quite different role in the formation and current understanding of the project among the participants. Twisting the perspective from the donor/initiator to the recipient discourse, SESAME is also understood as a response to the on-going exclusion and discrimination of Arab researchers in a western dominated international science community ^121^. It has been reported in several talks and interviews that it has proven difficult for Arab researchers to get their proposals accepted in order to be allowed to conduct research in the advanced European facilities. By contrast, Israeli researchers are much more likely to get access to western facilities. In contrast to most Arab research communities, they have direct access for example to ESRF and Elletra Sincrotrone Trieste in Italy ^122^. The positive effects of a synchrotron (community) within the region might therefore not be self-evident for Israeli researchers from the start but it would be important to secure their involvement ^123^. At the same time, this also makes them an important member of SESAME. Yet again, it can be more difficult for Israelis to reach a facility that is located a couple of kilometres right beside their country than to fly to European synchrotrons. For Israeli researchers it is not easy to cross the border to Jordan on their way to SESAME ^124^.

To sum it up, while the value of SESAME has often been deemed to lie in its peace-making capacity in the region, this is mainly from the point of view of the international press and of the international community. It might be just as much considered a needed redress of a lack of synchrotron technological development in the region and maybe discrimination and exclusion of Arab researchers in the international arena. In that sense, the topic of ex/inclusion plays a vital role on the level of defining SESAME’s purpose and core identity. 

### 5.8 Gender

Women made substantial contributions to SESAME and it is fair to say that SESAME would not exist in its current form without the input of a number of individual female scientists. One of the two beamline scientists in charge is Gihan Kamel, a woman from Egypt. She joined SESAME as early as 2005 and has pushed forward the construction of the IR beamline from the beginning. Unfortunately, she has also been the only woman working permanently in the facility for a long time and she had to deal with difficulties because she is a woman working full-time ^125^. Another woman, who has been part of the evolution of SESAME almost from the beginning, is Zehra Sayers. She was a driving force for SESAME in the function of the long-standing chair of the Scientific Advisory Committee (SAC) (for more details on her outstanding role and contribution see Section 4.4).

Apart from the key contributions by individual women, women seem to be generally more active and are better represented (at least currently, 2019) than men in the current users group ^126^: The first official experiment that was ever conducted at SESAME and at the XAFS/XRF beamline was led by Kirsi Lorentz, a woman from Cyprus. The second official experiment, conducted at SESAME and the first one officially conducted at the IR beamline, was also led by a team of female researchers from Egypt, Kairo University. It would be interesting to know more about the reasons for that finding. Yet it generally matches the fact that women researchers are well represented in scientific institutions in many Arab countries (e.g. Tunisia or Egypt) often contradicting widespread beliefs among Europeans ^127^. On average, women are better represented statistically in research institutions in Arab countries than in North America or Western Europe.

At the directorate level, there are currently no women at all.

## 6. Conclusion and recommendations

SESAME is a unique science collaboration and science diplomacy effort in the Middle East. Its core ambition is to operate an international state-of-the-art synchrotron radiation users facility in Jordan that is accessible to scientists from the member countries Cyprus, Egypt, Iran, Israel, Pakistan, Palestine, Turkey and Jordan. In that, it wants to advance scientific and technological development in the region and reverse brain drain. Being the first synchrotron in the Middle East region it maintains enormous potential with regards to furthering individual disciplines and research fields as well as strengthening the community of researchers in the region as a whole. SESAME also constitutes a science diplomacy effort with the aim of creating new links and intercultural understanding between scientists in this conflict affected region. The EU and European actors have played (and still play) an important role in many regards and on several layers.

SESAME is mainly an effort of scientific actors, namely the international synchrotron community that was institutionalized with the strong support of UNESCO. It was therefore brought into life first of all by the dedication of individual scientists and the institutional support of the broader synchrotron user facilities community, namely national research institutes and science associations. Science associations and synchrotron facilities from Europe played a crucial part in this, both at a national and supranational level, namely CERN, ESFR, the Helmholtz Foundation (with DESY and BESSY), SOLEIL and many others. These facilities have provided expertise, experience, components and training. SESAME is in large part a result of their enterprise. Having said this, the European Union has also made major contributions to SESAME. By contrast, European Member States have not been explicitly active or supportive in general.

In the case of SESAME, the EU seems to have gotten many things right. It has not only provided considerable financial resources (in the order of EUR 10 to 20 million) throughout the last 15 years, but this has happened also in an almost integrated approach. It enabled the allocation of resources (also from other national donors) in close cooperation with the executives and with regards to the particular needs of the facility. Resources were spent on the procurement and commissioning of technical components and even full beamlines (most recently the BEATS Project). It has funded the construction of an on-grid solar power plant in Jordan that satisfies the high electricity consumption of the particle accelerator (in that, SESAME is the first synchrotron in the world that has gone green) and in that the EU has made a major investment in the future of the synchrotron facility as well as in environmental friendly energy consumption. Thirdly, the EU has supported the development of human capacity and networks. Most importantly in that regard, the EU provided funds for OPEN SESAME, a Horizon 2020 consortium consisting of 10 European synchrotron facilities and science associations to train and advise SESAME staff and users and to connect them with each other. On top of that, the consortium aims to develop a strategy to reach out to scientists in the region, develop the users community and promote the purpose of SESAME in the regional industry. Investments (not only those of the EU, of course) are very slowly starting to pay off. The year 2019 marks the first year in which the facility is open to users on an almost regular basis and research is being conducted. Apart from the full operation of the beamlines, SESAME has brought together hundreds of (mostly young) scientists from the region in training and users meeting, and scientists from the Middle East and beyond have collaborated closely in the set-up of the facility in recent years. In a few instances it has in fact “reversed brain drain” and caused “brain circulation” ^128^. Finally, it should not be forgotten that SESAME receives credit for its support in the region. Staff and users of SESAME are well aware of the EU’s contributions and this is interpreted as a neighborhood policy, though a strategic and self-interested motivation is identified.

One of SESAME’s main obstacles is the little cooperation that happens at the member level. This refers to the fact that researchers/users communities at the national level are not developed and systematically organized yet. This makes it hard for SESAME to reach out to the potential users communities more effectively ^129^. Organizing (or helping to organize) users communities could also be a task for the national governments of the member states, such as approaching universities and science communities within their countries. The biggest diplomatic effort still remains in securing the yearly budget. SESAME has to continually struggle with the financial situation. Again, this is most probably a topic that also needs to be negotiated among the member countries and requires the commitment of political elites within the countries. SESAME has enjoyed wide institutional and symbolic support on the international political and scientific level to this day (e.g. by UNESCO, the European Union, national and international research centers such as CERN and the majority of European synchrotrons) that has equipped the project with international backing. Among others, the European Commissioner for Research (2014–2019), Carlos Moedas has been a strong proponent of the project.

Paradoxically, SESAME will be most effective in its science diplomacy effort if it does not try to be a science diplomacy effort at all costs. It should continue to put the focus on its scientific core purpose: providing a research facility and advancing the researchers (users) community in the region. The public narration and articulate expectation of SESAME as purposefully bringing people together and building bridges has been interpreted at times as a political agenda that is not always easy to read for everyone and can be interpreted as standing in opposition to scientific objectives. It has raised suspicion and resistance among the fairly diverse groups of researchers. Furthermore, the specific member country constellation – which is based on its science diplomacy ambition to comprise politically adverse countries – seems to have complicated the negotiation of (financial) support and commitment considerably. It has also made it difficult to attract new member countries. In the worst case, if SESAME showcases an overly explicit ambition of overcoming difference and peacemaking, it might just attract the staging of political conflicts. This scenario is unlikely from a current point of view, but should be considered. Maintaining an explicit science diplomacy ambition or not is independent from the fact, that people from different backgrounds do (and will) meet and overcome cultural biases while working at SESAME anyways. The science diplomacy narrative might have earned SESAME support within the international political community and might have propelled even more dedication by the international synchrotron community. We do not know this. Yet, SESAME was modeled on the example of CERN to foster international collaboration and development on scientific grounds. And if SESAME is to follow the example of CERN, this means that it has to continue to consolidate scientific effort before anything else. SESAME and staff should be supported in exactly this effort (they will pursue this effort anyway, we have no doubt about that).

## Data availability

Parts of the data basis for this study were generated through desk research in publicly available resources. These resources have been cited and can be assessed. Data that has been collected through interviews and observations on our research visit can not be made publicly available because we have granted anonymity to our research subjects. Interviewees did not give permission for their transcripts to be shared. Our informed consent sheet, signed by both us and interviewees, commits us to protecting their privacy and anonymity, stating that “personal details such as name, phone number, address and email address etc. will not be revealed to people outside the project”. Due to the specificity of the provided information and the small group of actors in the field we are unable to sufficiently anonymize transcripts. Aggregating information that was provided in individual transcripts would allow anyone to reconstruct the interviewees’ identities.

Please contact Charlotte Rungius ( charlotte.rungius@hu-berlin.de) in order to request access to the data. The conditions under which access will be granted are: 1, that the requested material is not protected by a confidentiality agreement; 2, that the material is to be used for unbiased, academic, non-profit academic purposes only; 3, that any direct quotation from the material in something intended for publication requires advance pre-approval; and 4, that any published use of the material include an acknowledgement stating the source of the material as (S4D4C project). The request for material should be accompanied by an explanation of the research being undertaken for which the data is needed and how it will be used, a complete CV of the person requesting the data and the CV of any others who will be given access to it, and a confirmation that the person has no conflict of interest and will not share the provided data with any third parties.

## Footnotes

^1^ S4D4C (“Using Science for/in Diplomacy for addressing global Challenges”) is a research project funded by Horizon 2020. The overall aim of S4D4C is to support current and future European science diplomacy for the benefit of European capacities, EU foreign policy goals and especially the development of solutions for global challenges.

^2^ Royal Society (2010): New Frontiers in Science Diplomacy: Navigating the Changing Balance of Power. London: Science Policy Centre, The Royal Society.

^3^ Ruffini, P.-B. (2017): Science and Diplomacy. A New Dimension of International Relations. Springer International Publishing.

^4^ We would like to express our deep-felt gratitude to the members and associates of SESAME for the support, trust and willingness to share their perspectives and experiences with us, anonymity granted.

^5^ The location was chosen due to the fact that scientists from all other member countries could travel to Jordan without restrictions, while this was not the case for most of the other member countries.

^6^ Interview#3

^7^ Owen, R., J. Juanhuix, M. Fuchs (2016): Current advances in synchrotron radiation instrumentation for MX experiments. In: Archives of Biochemistry and Biophysics, 2016, 602, pp. 21–31.

^8^ To get a more expert impression about the functioning of synchrotrons, please refer to the various introductory material online. On SESAME specifically please refer to http://www.sesame.org.jo/sesame_2018/about-us/information-material

^9^ The map shows the distribution of synchrotrons that run macromolecular crystallography (MX). MX is a specific synchrotron technology that operates in the X-ray spectrum and allows to study proteins and viruses. MX accounts for only about half of the existing synchrotron facilities worldwide, but represents a fair proxy for the distribution of operational synchrotrons worldwide. cf. Owen, R., J. Juanhuix, M. Fuchs (2016): Current advances in synchrotron radiation instrumentation for MX experiments. In: Archives of Biochemistry and Biophysics, 2016, 602, pp. 21–31.

^10^ Interview#3; Smith, C. L. (2012): Synchrotron Light and the Middle East: Bringing the Region’s Scientific Communities Together Through SESAME. In: *Science & Diplomacy*, 1(4).

^11^ Owen, R., J. Juanhuix, M. Fuchs (2016): Current advances in synchrotron radiation instrumentation for MX experiments. In: Archives of Biochemistry and Biophysics, 2016, 602, pp.

^12^ Smith, C. L. (2018): Science Beyond Boundaries: SESAME and the International Cooperation. In: S. Abousahl, W. Plastino (Eds.): International Cooperation for Enhancing Nuclear Safety, Security, Safeguards and Non-proliferation–60 Years of IAEA and EURATOM, pp. 175–185. Berlin, Heidelberg: Springer Verlag.

^13^ Stein, J. A. (2002): Science, technology and European foreign policy: European integration, global interaction. In: Science and Public Policy. 29(6), December 2002, pp. 463–477. ; Krige, J. (2005): Isidor I. Rabi and CERN. In: Physics in Perspective, June 2005, Volume 7, Issue 2, pp. 150–164.

^14^ SESAME (2010): An international centre for research and advanced technology under the auspices of UNESCO. Retrieved from: SESAME website

^15^ Smith, C. L. (2012): Synchrotron Light and the Middle East: Bringing the Region’s Scientific Communities Together Through SESAME. In: Science & Diplomacy, 1(4).

^16^ Lightsources.org: Light sources of the world. Retrieved from: https://lightsources.org/lightsources-of-the-world/

^17^ SESAME: Members and Observers of SESAME. Retrieved from: http://www.sesame.org.jo/sesame_2018/about-us/members-sesame

^18^ All interviews were conducted by Dr. Tim Flink (male) and Charlotte Rungius M.A., B.Sc. (female) both in the role of researchers in S4D4C. Both interviewers were employed at the DZHW (German Centre for Higher Education Studies and Science Research) at the time of the study. Additionally, Tim Flink was permanently associated with the Humboldt Universität zu Berlin. Both interviewers were practically experienced and had academic training in conducting interviews for the purpose of generating qualitative social research data. The role and occupation of the interviewers, the purpose of the study as part of S4D4C’s aims and ambitions, the specific research interest as part of the case study and contact information (website link, e-mail addresses, in some cases the S4D4C brochure if available) were revealed and/or handed over to the interviewees before the interview in written form (informed consent sheet and S4D4C information leaflet). Contact was established through email or in person at the users meetings. In both cases the research interest as the motivation to establish contact was stated clearly. There was no prior relationship to any of the interviewees. None of the approached participants refused to participate or dropped out. Repeat-interviews have not been conducted. The interviews did not strictly follow our interview guide in order to allow a natural conversation and information flow. Our interview guide was composed of the following six sections: I) function and responsibility at SESAME, II) biography and motivation, III) mission and impact of SESAME, IV) governance, funding and support (including the role of the EU), V) mode of operation on site, VI) outlook.

^19^ Setting of data collection: All interviews have been conducted in a professional environment: either at the offices of interviewees, the conference venue or at the research facility. No one else besides the interviewers and interviewees was present during the interviews, however those interviews that were conducted in the common spaces of the conference venue could not provide a completely confidential setting.

^20^ Schopper, H. (2017): The light of SESAME: A dream becomes reality. In: RIVISTA DEL NUOVO CIMENTO. 40(4), pp. 199–239.

^21^ Smith, C. L. (2018): Science Beyond Boundaries: SESAME and the International Cooperation (11 pages)

^22^ Interview#4

^23^ Khan, S. A. (1999): Salam’s bright idea. In: Physics World (Letters to the Editor). 12(11), p. 15.

^24^ Schopper, H. (2017): The light of SESAME: A dream becomes reality. In: RIVISTA DEL NUOVO CIMENTO. 40(4), p. 15

^25^ SLAC is short for “Stanford Linear Accelerator Center” and it is a U.S. Department of Energy (DOE) Office of Science laboratory operated by Stanford University.

^26^ DESY is short for “Deutsches Elektronen Synchrotron” and is located in Hamburg, northern Germany.

^27^ In operation from 1982 until 1999, the West Berlin based BESSY I (Berlin Electron Storage Ring Company for Synchrotron Radiation) was Germany’s first electron storage ring facility HZB (2019): Die Historie der Berliner Elektronen-Speicherring Gesellschaft für Synchrotronstrahlung (BESSY). Retrieved from: https://www.helmholtz-berlin.de/zentrum/forschungszentrum/campus/historie/index_en.htmlas accessed 15 June 2021.

^28^ Schopper, H. (2017): The light of SESAME: A dream becomes reality. In: RIVISTA DEL NUOVO CIMENTO. 40(4), pp. 199–239. ; Interview#3, 2018

^29^ Einfeld, D., S. S. Hasnain, Z. Sayers, H. Schopper, H. Winick (2004): SESAME, a third generation synchrotron light source for the Middle East region. In: Radiation Physics and Chemistry. 71(3–4), p. 694. https://doi.org/10.1016/j.radphyschem.2004.04.130

^30^ Schopper, H. (2017): The light of SESAME: A dream becomes reality. In: RIVISTA DEL NUOVO CIMENTO. 40(4), p. 201.

^31^ Ibid.

^32^ Ibid. p. 200–201.

^33^ Schopper, H. (2017): The light of SESAME: A dream becomes reality. In: RIVISTA DEL NUOVO CIMENTO. 40(4), p. 205.

^34^ Ibid. p. 206.

^35^ The 12 members were Armenia, Bahrain, Cyprus, Egypt, Greece, Iran, Israel, Jordan, Morocco, Oman, Palestinian Authority and Turkey. After the determination of the site’s location, Armenia, Morocco and Oman left the interim council (Schopper 2017, 206).

^36^ Einfeld, D., S. S. Hasnain, Z. Sayers, H. Schopper, H. Winick (2004): SESAME, a third generation synchrotron light source for the Middle East region. In: Radiation Physics and Chemistry. 71(3–4), p. 695

^37^ Khan, S. A. (2003): The Middle East Synchrotron is Launched. In: AAPPS Bulletin (News), 13(2), pp. 35–36.

^38^ Schopper, H. (2017): The light of SESAME: A dream becomes reality. In: RIVISTA DEL NUOVO CIMENTO. 40(4), pp. 199–239.

^39^ Ibid. p. 212.

^40^ Ibid. p. 212–213.

^41^ Ibid. p. 213.

^42^ Ibid. p. 214

^43^ Ibid. p. 207–208

^44^ Ibid. p. 214–215

^45^ Ibid. p. 221–222

^46^ Smith, C. L. (2018): Science Beyond Boundaries: SESAME and the International Cooperation, p. 178.

^47^ Schopper, H. (2017): The light of SESAME: A dream becomes reality. In: RIVISTA DEL NUOVO CIMENTO. 40(4), p. 210.

^48^ Einfeld, D., S. S. Hasnain, Z. Sayers, H. Schopper, H. Winick (2004): SESAME, a third generation synchrotron light source for the Middle East region. In: Radiation Physics and Chemistry. 71(3–4), p. 210. Initial observers were Oman, UAE, Morocco. Later Cyprus and Iran joined as members, as well as France, Germany, Italy, Japan, Kuwait, Portugal, Russia, Sweden, UK, USA and EU as observers (Schopper 2017, 210).

^49^ Khan, S. A. (2003): The Middle East Synchrotron is Launched. In: AAPPS Bulletin (News), 13(2), pp. 35–36.

^50^ SESAME (2010): An international centre for research and advanced technology under the auspices of UNESCO.

^51^ Smith, C. L. (2018): Science Beyond Boundaries: SESAME and the International Cooperation.

^52^ Ibid. p. 179.

^53^ Ibid. p. 179.

^54^ Ibid. p. 181.

^55^ SESAME (2010): An international centre for research and advanced technology under the auspices of UNESCO.

^56^ Interview#4

^57^ Interview#4 and Interview#5

^58^ Interview#3

^59^ https://www.sesame.org.jo/about-us/sesames-structure/organization-chart-of-sesame

^60^ Interview#4

^61^ Paolucci, G. (2016): SESAME: A Concrete Science for Peace Project in the Middle East. p. 22. Retrieved from: http://www.namesnetwork.org/Names2016/Attachments/Speakers/2017012310403451137_GP.pdf

^62^ SESAME/DEADSEA/25-C/14-13

^63^ SESAME/AMMAN/30-C/17-03

^64^ https://www.youtube.com/watch?v=dohcpJddNGQ, time stamp 5:53.

^65^ Ibid. time stamp 10:17.

^66^ We have found no evidence that the Advisory Committee for Beamlines is currently active or in place.

^67^ Interview#4, Interview#5

^68^ Users are organized in the SESAME Users’ Committee. This committee consists of one representative for each member country ( http://old.sesame.org.jo/sesame/users/sesame-users-committee-suc/membership.html)

^69^ Interview#5

^70^ Interview#5

^71^ SESAME/AMMAN/27-C/15-14

^72^ There are almost no media articles that do not introduce SESAME with a reference to CERN.

^73^ Smith, C. L. (2018): Science Beyond Boundaries: SESAME and the International Cooperation, p. 180.

^74^ Ibid. p. 179. Just like SESAME, CERN is organized as an international research organization that is principally independent from the EU. Most member countries are European, but not all are part of the EU such as Norway and Switzerland. The EU has observer status at CERN.

^75^ Interview#3

^76^ “The EU has been very supportive. They deserve a lot of credits.” (Interview#3)

^77^ Interview #5; „They gave us human capacity. They gave us the opportunity for training for our staff and for training the users community. They gave us one beamline and they gave us funds for our operational. It is really an integrated approach. They gave us a lot of opportunities. So I think that this integrated approach is really successful.” (Interview#5)

^78^ Interview#5

^79^ Interview#5

^80^ More details about “Open SESAME” are covered in section 5.1

^81^ (Interview#5)

^82^ (Interview#4)

^83^ In 2017, 16 European particle accelerator facilities have formed a common umbrella organization called LEAPS in order to “jointly develop methods, instruments, and data-management approaches; avoid duplication; and communicate with policymakers and funding agencies.” ( https://physicstoday.scitation.org/do/10.1063/PT.6.2.20190123a/full/)

^84^ Interview#4

^85^ Taiwan started with a 1.3 GeV synchrotron and pushed it to 1.5 GeV. Therefore it could not do the high energy X-rays. The design was proposed by western physicists (H. Winick was one of them). They later recognized that they were too modest with regards to the proposed performance layout (Interview#3).

^86^ Interview#3

^87^ Paolucci, G. (2016): SESAME: A Concrete Science for Peace Project in the Middle East. Presentation slides. Retrieved from: http://www.namesnetwork.org/Names2016/Attachments/Speakers/2017012310403451137_GP.pdf

^88^ Ibid.

^89^ The Abdus Salam International Centre for Theoretical Physics (ICTP)

^90^ Ibid.

^91^ DIAMOND is the biggest national synchrotron user facility in the UK.

^92^ Al-Zoubi, A. (2018): Diamond-Sesame Fellowship Grant: Supporting Scientific Knowledge Exchange. 16 ^th^ SESAME users meeting.

^93^ Ibid.

^94^ Interview#4

^95^ Interview#4

^96^ Interview#5

^97^ Proposals are submitted through SESAME’s online portal: https://sup.sesame.org.jo/pls/vuo/guest.startup

^98^ Interview#1

^99^ Scientific priorities do not directly and not necessarily translate into political priorities. Researchers and potential users of SESAME have been called upon to approach their respective governments and exert political pressure at home. It remains an open question, to which extent this can yield successful results and serve as a promising leverage.

^100^ Smith 2012; Interview#4

^101^ Interview#4

^102^ Interview#4

^103^ Interview#4

^104^ Interview#4

^105^ At the same time, it has to be taken into account that otherwise it would not have been realized at all.

^106^ Daresbury and LURE (Laboratoire pour l’Utilisation du Rayonnement Electromagnétique)

^107^ Interview#1

^108^ Interview#4

^109^ One interviewee reported: “At that time it was like a shock for me: Why are we getting the garbage?” (Interview#1)

^110^ Interview#1 and Interview#2

^111^ Interview#2

^112^ Interview#1; Interview#2

^113^ Interview#2

^114^ Interview#1

^115^ Interview#1

^116^ “I think you have noticed that when Ron started his talk, people were pulling out. [...] Because he is from Israel. We do not have to listen to him talking about crystals. We know crystals. We have the same knowledge. We do not have to listen to him. Why? The question is why! Because those scientists do not also stand up against their society and say ‘Hey, people, you politicians, you are doing wrong things in the regions. You are not respecting the rights of the people in the region.’” (Interview#1)

^117^ Interview#1

^118^ Kamel, G. (2018): SESAME Synchrotron Light Source, Why in the Middle East? In: L. Maiani *et al*. (eds.): International Cooperation for Enhancing Nuclear Safety, Security, Safeguards and Non-proliferation–60 Years of IAEA and EURATOM. Springer Proceedings in Physics 206, p. 188. https://doi.org/10.1007/978-3-662-57366-2_27

^119^ Ibid. p. 188.

^120^ Interview#2

^121^ “We or most of the scientists here in the region, they usually do not have collaboration with scientists from Germany or France or whatever. It is like there is a gap. We cannot cover it.” (Interview#1)

^122^ Interview#4

^123^ Interview#4

^124^ Interview#4

^125^ Interview#4

^126^ „We have really a lot of good scientists that are women. It is not difficult to get a good gender balance.” (Interview#5)

^127^ Plackett, B. (2018): Behind the Numbers: Arab Women in Research, In: Al-Fanar Media, 6 August 2018. Retrieved from: https://www.al-fanarmedia.org/2018/08/behind-the-numbers-arab-women-in-research/

^128^ Kamel, G. (2018): SESAME Synchrotron Light Source, Why in the Middle East? In: L. Maiani *et al*. (eds.): International Cooperation for Enhancing Nuclear Safety, Security, Safeguards and Non-proliferation–60 Years of IAEA and EURATOM. Springer Proceedings in Physics 206

^129^ OPEN-SESAME (2018): Recommendations on maximizing the socio-economic impact and securing the long-term sustainability of SESAME. Grant agreement n. 730943.

