# Supplementary material for: SESAME – a synchrotron light source in the Middle East: an international research infrastructure in the making
Source: Open Res Eur. 2022 Feb 4;1:51. Originally published 2021 May 17. [Version 2] doi: 10.12688/openreseurope.13362.2 (PMC10445914; doi:10.12688/openreseurope.13362.2)
Supplement: Peer review history

# Peer review: Reviewer response for version 2

**DOI:** 10.21956/openreseurope.15603.r28519

**Published:** 2022-02-21

**Authors:** Gihan Kamel

**Affiliations:** 1 SESAME (Synchrotron-light for Experimental Science and Applications in the Middle East), Allan, Jordan; 2 Department of Physics, Faculty of Science, Helwan University, Cairo, Egypt

The authors’ revision regarding the raised case study-related concerns was adequate and convincing. In addition, the suggestions provided in order to enhance the manuscript quality, were also considered.

Is the case presented with sufficient detail to be useful for teaching or other practitioners?

Yes

Is the work clearly and accurately presented and does it cite the current literature?

Partly

If applicable, is the statistical analysis and its interpretation appropriate?

Partly

Are all the source data underlying the results available to ensure full reproducibility?

No source data required

Are the conclusions drawn adequately supported by the results?

Yes

Is the background of the case’s history and progression described in sufficient detail?

Yes

Reviewer Expertise:

NA

I confirm that I have read this submission and believe that I have an appropriate level of expertise to confirm that it is of an acceptable scientific standard.

# Peer review: Reviewer response for version 2

**DOI:** 10.21956/openreseurope.15603.r28518

**Published:** 2022-02-11

**Authors:** Zehra Sayers

**Affiliations:** 1 Biological Sciences and Bioengineering, Faculty of Engineering and Natural Sciences, Sabancı University, Istanbul, Turkey

I think the changes that have been made in the article are appropriate.

Is the case presented with sufficient detail to be useful for teaching or other practitioners?

Yes

Is the work clearly and accurately presented and does it cite the current literature?

Partly

Are all the source data underlying the results available to ensure full reproducibility?

No source data required

Are the conclusions drawn adequately supported by the results?

Yes

Is the background of the case’s history and progression described in sufficient detail?

Yes

Reviewer Expertise:

NA

I confirm that I have read this submission and believe that I have an appropriate level of expertise to confirm that it is of an acceptable scientific standard.

# Peer review: Reviewer response for version 1

**DOI:** 10.21956/openreseurope.14432.r27432

**Published:** 2021-09-28

**Authors:** Gihan Kamel

**Affiliations:** 1 SESAME (Synchrotron-light for Experimental Science and Applications in the Middle East), Allan, Jordan; 2 Department of Physics, Faculty of Science, Helwan University, Cairo, Egypt

The case study presented by Runguis *et al.*, examines the establishment and the operational development phases of the SESAME synchrotron light source. It also provides a comparative investigation to show relationships between SESAME and other similar facilities. It is clear that the core focus of the report is on the science diplomacy axis. However, moving from one section to another gives the strong impression that it also targets the functionality of SESAME as an intergovernmental research Centre, as well as, its main role as a unique endeavour of science and technology in the Middle East and neighbouring regions. The report, once amended, will for sure provide the community with crucial information necessary to know about the brief history of SESAME which is important for the continuous success of the SESAME facility in general and its users’ community from all-over the world in particular. I found it very constructive in general, and it proved to be neutral and transparent in confronting the challenges SESAME is still facing but also nicely presenting the positive aspects and outcomes of such a unique facility of what concerns the scientific merits and values.

 The manuscript is generally well structured, but sometimes the authors anticipate statements before they have to appear in the text and/or repeat some statements in different sections. The report needs some improvements and clarifications before one can recommend it for indexing. Therefore, I approve it with reservations. I would advise a consequential detailed study on the individual challenges those may threaten the stability, the fate, and the progress of SESAME.

 An annotated version of the manuscript with further comments and corrections can be found here: 

https://openreseurope.s3.amazonaws.com/supplementary/13362/42a083cc-3cab-4bf4-8a2b-896fcd16e90b.pdf

Is the case presented with sufficient detail to be useful for teaching or other practitioners?

Yes

Is the work clearly and accurately presented and does it cite the current literature?

Partly

If applicable, is the statistical analysis and its interpretation appropriate?

Partly

Are all the source data underlying the results available to ensure full reproducibility?

No source data required

Are the conclusions drawn adequately supported by the results?

Yes

Is the background of the case’s history and progression described in sufficient detail?

Yes

Reviewer Expertise:

NA

I confirm that I have read this submission and believe that I have an appropriate level of expertise to confirm that it is of an acceptable scientific standard, however I have significant reservations, as outlined above.

# Peer review: Reviewer response for version 1

**DOI:** 10.21956/openreseurope.14432.r27430

**Published:** 2021-09-16

**Authors:** Zehra Sayers

**Affiliations:** 1 Biological Sciences and Bioengineering, Faculty of Engineering and Natural Sciences, Sabancı University, Istanbul, Turkey

The paper by Runguis *et al*., is a detailed study of the complicated process of the SESAME laboratory’s establishment. It also attempts to provide an account of the functioning SESAME facility during its early operational days at the end of 2018 and early 2019. The authors approach the SESAME project from a science diplomacy perspective but also capture well the spirit of the project as a science driven case based on an instrument (facility) that has to function at world standards in order to succeed. They provide an interesting and frank discussion on its limitations and short comings particularly from the point of view of inclusion and exclusion of actors. There is also a good discussion of various instruments that international scientific community have generated to support SESAME. I consider this paper as a worthy addition to the literature on SESAME. I recommend its indexing after the points listed below are addressed*.

 Having said this, I would like to draw attention to two topics which I believe merit further consideration than given in the manuscript. One is SESAME’s intergovernmental funding structure which I think is unique among user facilities in the world. Secondly, the authors might have further investigated the cohesion among the members of the SESAME Council and the individual scientists who make the laboratory work. This feature is worth elaborating further, given the diversity of the countries involved and given that the project was developed by individual scientists and not as an intergovernmental venture. Where do the expectations of scientists and those of the Council meet? What are the factors affecting the ownership feeling of the member countries? How can the spillover scientific effects of SESAME in the individual member countries be assessed? Finally, what additional means, other than producing excellent science, can SESAME use to support acceptance of science in the region?

 *Specific points are listed below:

 Suggested changes are given comment numbers and are highlighted green in the pdf of the manuscript. This file can be found here: https://openreseurope.s3.amazonaws.com/supplementary/13362/4faf5d89-42f8-4f2b-ac8b-d067f952a062.pdf

 Page numbers are also indicated below.

Page 3.

 1. Insert “operational” instead of “installed”.

 2. Insert “project was conceived” instead of “was established”. Because “SESAME” which is the laboratory was established in 2003/4.

 3. Could the word “primarily” reduce the emphasis made later on the importance of science?

Page 4.

 4. Replace “international” with “intergovernmental”

 5. “A synchrotron….” This is somewhat inadequate description of a synchrotron giving the impression that it is only for biology. One can say “..of matter such as viruses, crystals, proteins, semiconductors, archaeological artifacts etc”.

 6. “device” should be” devices”.

 7. Caption Figure 1: Change to “Schematic outline of the components of SESAME synchrotron. Experimental stations branch out from the storage ring”. Reference for this figure should be given. (p. 5)

 8. Caption Figure 2: Change to “Photo of the experimental Hall December 2018”. Reference for this figure should be given. (p. 5)

 9. I do not know of synchrotrons that are intergovernmentally funded other than SESAME.

 10. Footnote 9 and Figure 3: (1) As mentioned in the footnote this map shows only facilities with MX beamlines, it can be a bit misleading with about 20 facilities visible and also SESAME is not on this map. A better alternative would be the map from lightsources.org/light-sources-of-the-world/ which also shows SESAME.

 If they insist in using this map I suggest the to change the caption as given in the following:

 The map shows the distribution of synchrotrons that run macromolecular crystallography (MX) [insert “beamlines”]. MX is a specific synchrotron [technologyà technique”]  that [operatesàuses”] in the X-ray[insert “s”] spectrum and allows to study proteins and viruses [insert ”in atomic detail”].[Insert “Facilities with”] MX “beamlines” accounts for only about half of the existing synchrotron facilities worldwide, but [insertà”the map gives an idea about”] represents a fair proxy for the distribution of operational synchrotrons worldwide.

Page 6.

 11. Insert “Palestine” instead of “Palestinian authority”.

 13. Figures should be numbered in an ascending order in accordance with their appearance in the text.

 14. Insert “comes only at” instead of “scales to”.

 15. Insert “among” instead of “between”.

 16. Insert “laboratory” instead of “research site”.

 17. It is not clear what “SESAME 2010” means. The full address of the website should be given each time it is cited.

Page 7.

 18. A reference should be given for the source of Figure 4.

 19. “following section” and “(Section 2.1)” are contradictory since Section 2.1 was the previous section.

Page 8.

 20. Figure 5 is not up-to-date because already operational facilities e.g. SOLEIL is shown in gray.

Page 9.

 21.  This sentence is hard to understand.

 22. Insert “Chairs” instead of “Directors”

 23. The two highlighted sentences sound contradictory.

 24. Insert “Director” instead of “Acting Director-General”.

Page 10.

 25. Abdus Salam did not promote SESAME because SESAME did not exist then. He promoted the idea of an international laboratory in the Middle East. Text should be changed accordingly.

 26.  Footnotes 25 and 26: one may also refer to the websites.

 27. It is not clear what “HZB (2019)” means.

Page 11.

 28. I suggest removing “revived Salam’s vision and” since we do not know exactly was his vision was.

 29. This statement is too vague and it is not clear which countries are involved. I suggest removing it.

 30. Remove “organization…UNESCO” insert “center”.

 31. Remove “Morocco” insert “Pakistan”. Remove “Morocco and Oman”

Page 12.

 32. Replace “Russia and Sweden” with “International Interim Council members, UNESCO, ICTP and Japanese scientists” Source for this is SESAME Strategic Plan 2010-2014.

 33. Replace “set up” with “developed or prepared”.

 34. Replace “UNESCO rules” with “Statutes of SESAME”.

 35. Here there is a need to insert an additional phrase:  “(for further members/observers see Footnote XX)

 36. Remove “Initial observers were Oman, UAE, Morocco”.

 Insert an additional Footnote.

 Footnote XX: Later Cyprus and Iran joined as members. In the early stages (International Interim Council period) Armenia, Greece, Oman and UAE made contributions to the budget.  Germany, Italy, Japan, Kuwait, Morocco Russian Federation, Sudan, Sweden, UK and USA were initial observers.

 37. Remove “each contributed USD 5 million to the capital budget” ” insert ”each pledged to contribute USD 5 million to the capital budget. Although others paid, Iran could not make the payments due to the international sanctions. ”

 38. Replace “funded CERN with the same sum to” with “through CERN provided 5 million Euro to...”

 39. Complete references to footnotes 50 and 51.

Page 13.

 40. replace “spectrum;IR and XFAS” with “range; IR and XFAS/XRF beamlines)”

 41. Replace “up to 18 more” with “more than 18”

 42. Replace “two.........a soft X-ray beamline” with ”Afterwards the materials science beamline has become operational with two more being under construction (soft X-ray spectroscopy (HESEB) and tomography (BEATS). Three more (crystallography (MX), small angle X-ray scattering (SAXS) and soft X-ray spectroscopy (TXPES) beamlines are also being designed”.

 43. What is the cut-off date for his article? The guest house is finished since December 2019.

 44. Remove “which has not yet been realized”

 45. Remove “operational” because it is not clear what it means in this context.

 46. Footnote 55 needs to be completed.

 47. Replace “Palestinian authority” with “Palestine”

Page 14.

 48. Replace “due……contribution” with “possibly because its internal infrastructure was not yet sufficient”.  Please check the reference for the footnote 62. What is mentioned in this document is a decision and not a statement on the discord about the financial contribution.

Page 15.

 49. Figure 8: this table is hard to understand would it be possible to change?

 Combining information from different sources (the Annex to SESAME Statutes, Strategic plan 2010-2014 and some early records), it is possible to say that initial list of countries that made a payment of *a kind* are: Armenia, Bahrain, Cyprus, Egypt, Greece, Iran, Israel, Jordan, Oman, Pakistan, Turkey and UAE. These can be considered as members of the International Interim Council.

 Those that later became the founding members (members of the Permanent Council) are: Bahrain, Cyprus, Egypt, Iran, Israel, Jordan, Pakistan, Palestine, Turkey.

 Early observers are: France, Germany, Italy, Japan, Kuwait, Morocco, Russia Federation, Sudan, Sweden, UK and USA.

Page 16.

 50. I ask to kindly remove this sentence. This statement is against the spirit of SESAME and there is no solid evidence to prove that this is the case.

 51. Remove “considerable” because this is not actually the case.

 51a. This sentence is not clear/correct. Representatives of countries are official envoys. Ambassadors to UNESCO do not have anything to the with SESAME.

 52. Insert “program” after ”development”.

 53. Replace “Hamburg” with “EMBL Hamburg Outstation on the synchrotron”.

 54. Replace “PhD scholar” with “postdoc”.

 55. Rewrite Footnote 66 as: Advisory Committee for Beamlines was merged with SAC in 2012.

Page 17.

 56. Replace “TAC” with “SAC”

 57. Replace “staff” with “scientists”

 58. The IAEA has been a major supporter of SESAME from very early on, since 2006, providing support for training and expert visits and deserves a mention on its own. I suggest to split the highlighted sentence to mention IAEA on its own. Suggestion: “The IAEA has been an important for SESAME providing scientific and financial support.  The Jordan Atomic Energy Commission has been providing necessary political backing in Jordan.”

 59. “(Smith 2018, 180)” needs to be taken out of here. In footnote 73 the full reference should be given.

Page 18.

 60. BEATS is not just in cooperation with ESRF but it is a consortium of 10 institutions in Europe: ESRF, ALBA, The Cyprus Institute, DESY, INFN, PSI, SESAME, Solaris, Elettra. ESRF is the institution leading the project.

 61. In this collaborative work staff of SESAME and other young facilities are involved in all the steps of setting up the beamline.

 62. CALIPSO is not geared towards supporting trips to SESAME. It is for supporting trips to any synchrotron facility in Europe.

 63. Remove “mainly”

Page 19.

 64. Replace “lend” with “lent”.

 65. Replace “the current staff” with “some of the current staff”.

 66. Remove “now”.

 67. I am not sure what is meant here. Maybe one should remove “and”.

 68. Replace “get” with “are provided”.

 69. Should be written in capitals.

 70. As mentioned in the beginning of this paragraph, SESAME has received different levels and types of support from many institutions for training. The authors have grouped the components of the funding landscape as national scientific societies, international organizations and “national” synchrotron facilities. I recommend removing “national”. ESRF can be moved to the group of synchrotrons, Portugal should be taken out of the synchrotron group. Scientific societies could include the consortium Physical Societies from USA and Europe. 

Page 20.

 71. Footnote 93: ibid for what?

 72. Remove “major and”. IAEA training efforts are also institutionalized

 73. Replace “usually…..facility)” with “in Jordan. Since 2001 only 4 took place in member countries including two in Cairo, one in Turkey and one in Isfahan”.

 74. Replace “beam time” with “beamtime”77

 75. Remove “EMIRA”

 76. Remove “BASEMA”

 77. There has been three calls for project proposals so far. The part that is highlighted can be rephrased (based on actual numbers recorded at SESAME) as follows:

 An early call in 2017 resulted in 36 proposals for the XAFS and 19 proposals for the IR beamlines. In the second call, 2018, the numbers went up to 60 for XAFS and 43 for the IR and 12 proposals from these came from scientists who got involved through OPEN SESAME schools. However, the facility was not ready soon enough for the created research demand. In 2018 the machine delivered 15 weeks of beam to the beamlines (used also for commissioning) and 18 proposals were allocated beamtime on XAFS and 6 on the IR beamlines. Due to the delays in the running of the beamlines, the original schedules could not all be maintained and confirmations could not be adhered to, which has created dissonances and frustration among the users’ community already.

Page 21.

 78. Replace “60,000” with “50,000”

 79. This sentence should be removed because Egyptian contribution was never frozen.

 80. Replace “other cases” with “another case”. There were sanctions only for Iran.

 81. “Germany, Japan, Sweden and Russia” should be taken out. Only Italy contributed financial resources.

Page 22.

 82. Insert “Switzerland, Germany” as well as UK and France.

 83. This statement is highly speculative impossible to prove.

 84. I find it inappropriate that a name is mentioned.

Page 23.

 85. Replace “between” with “among”.

 86. I could not confirm that this statement is correct.

Page 24.

 87. Gihan Kameel became a SESAME staff member in 2015.

 88. Replace “Kairo” with “Cairo”.

 89. After “SOLEIL” insert “SLS, ALBA, Elettra, DIAMOND“.

 90. Replace “10-20” with “17-18”. This is closer to the actual amount.

Page 25.

 91. Replace “Research” with “Research, Science and Innovation”.

 92. Replace “anyways” with “anyway”.

Is the case presented with sufficient detail to be useful for teaching or other practitioners?

Yes

Is the work clearly and accurately presented and does it cite the current literature?

Partly

Are all the source data underlying the results available to ensure full reproducibility?

No source data required

Are the conclusions drawn adequately supported by the results?

Yes

Is the background of the case’s history and progression described in sufficient detail?

Yes

Reviewer Expertise:

NA

I confirm that I have read this submission and believe that I have an appropriate level of expertise to confirm that it is of an acceptable scientific standard, however I have significant reservations, as outlined above.
